# Plastic Waste Conversion over a Refinery Waste Catalyst

**DOI:** 10.1002/anie.202104110

**Published:** 2021-06-15

**Authors:** Ina Vollmer, Michael J. F. Jenks, Rafael Mayorga González, Florian Meirer, Bert M. Weckhuysen

**Affiliations:** ^1^ Inorganic Chemistry and Catalysis, Debye Institute for Nanomaterials Science Utrecht University Universiteitsweg 99 3584 CG Utrecht The Netherlands

**Keywords:** Aromatics, coke formation, fluid catalytic cracking, plastic recycling, polypropylene

## Abstract

Polypropylene (PP) makes up a large share of our plastic waste. We investigated the conversion of PP over the industrial Fluid Catalytic Cracking catalyst (FCC‐cat) used to produce gasoline from crude oil fractions. We studied transport limitations arising from the larger size of polymers compared to the crude oil‐based feedstock by testing the components of this catalyst separately. Infrared spectroscopy and confocal fluorescence microscopy revealed the role of the FCC matrix in aromatization, and the zeolite Y domains in coking. An equilibrium catalyst (ECAT), discarded during FCC operation as waste, produced the same aromatics content as a fresh FCC‐cat, while coking decreased significantly, likely due to the reduced accessibility and activity of the zeolite domains and an enhanced cracking activity of the matrix due to metal deposits present in ECAT. This mechanistic understanding provides handles for further improving the catalyst composition towards higher aromatics selectivity.

## Graphical abstract

On the path to finding better catalysts for polyolefin plastic waste conversion to aromatics, we have found that a waste refinery catalyst performed better than the fresh catalyst with decreased accumulation of deactivating carbonaceous deposits. Fluorescence microscopy and vibrational spectroscopy has been used to elucidate the interaction of the polymer with the catalyst and to map the location and type of bulky aromatic species.

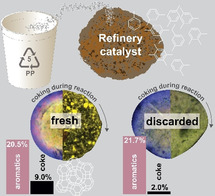

## Introduction

More than a century of polymer science and engineering has led to the development of plastics that are durable, lightweight and extremely versatile. Especially during the pandemic we are experiencing now, plastics have proven essential for hygiene,^[1]^ but our society also heavily depends on them for e.g., food preservation, construction materials, and electronics. The propensity of plastics to slowly degrade to form micro‐ and nano‐plastics, but not enough to be fully biodegradable means that plastic waste is a threat to the environment and human health.[^2^, ^3^]

Recycling plastics to products with the same exact properties is impossible via classical methods, such as melting and re‐extrusion, i.e., mechanical recycling.^[4]^ The plastic chemically changes during the process and perfect sorting is not (yet) feasible. Plastics versatility due to differences in monomer choice, polymer chain length and branching as well as additives, such as dyes and softeners, makes separating only one type of plastic extremely complex. With chemical recycling, the plastics are broken down to their chemical components, which circumvents some of the problems of mechanical recycling, albeit being more energy intensive.[^5^, ^6^] Ways to chemically recycle plastics have been researched at least since the 1960s and pilot plants were already operational in the 1990s,[^7^, ^8^] but the lack of regulation, like a carbon tax means that making new plastics from fossil resources is still very cheap and recycling has a hard time to compete. This is also largely because crude oil refinery infrastructure, leading to the production of ethylene, propylene as well as aromatics, exists and has worked very reliably for decades^[9]^ and the incentive to invest in new infrastructure for chemical recycling is still relatively low. Thus, it makes sense to explore chemical recycling routes that make use of existing oil refinery infrastructure, while other efforts are under way to transition to a fully circular economy. Such a chemical recycling process would either produce monomers for polymer makers directly, a naphtha drop‐in to be used in naphtha crackers, a pure stream of methylbenzene, or fuels like gasoline and diesel.

In the case of chemical recycling of polystyrene (PS), polymethylmethacrylate (PMMA) and polyethylene terephthalate (PET), monomers can be recovered, which can then be used to make new plastic with the same quality as the virgin material.[^10^, ^11^, ^12^, ^13^] For polyolefins, such as polyethylene (PE) and polypropylene (PP), monomer recovery is more difficult and arguably the easiest way to currently recover value from them is pyrolysis.[^14^, ^15^, ^16^, ^17^, ^18^, ^19^, ^20^, ^21^] However, with noncatalytic pyrolysis, a low value mixture of mostly cyclic alkanes and branched alkenes is recovered, as we will also show in this work.^[22]^ Indeed, heating the plastics under inert atmosphere to temperatures above 450 °C causes a random scission process via a radical pathway. With the addition of a catalyst the product scope changes towards a mixture of alkanes and methyl‐aromatics with the additional benefit that the process can operate at a lower temperature.^[23]^ Hence, the quest for catalytic upcycling routes for the recycling of plastics.^[24]^

Indeed when a catalyst material is provided, the reaction mechanism proceeds via the formation of carbenium ions^[24]^ and is somehow similar to what is well‐known from a crude oil refinery step, called Fluid Catalytic Cracking (FCC) in which Vacuum Gas Oil (VGO) is typically converted into gasoline.[^9^, ^25^, ^26^, ^27^] Gasoline consists of paraffins, olefins, cyclic alkanes, and aromatics in the range of C4‐C12.[^9^, ^28^] FCC units also produce a significant amount of propylene for PP production and other raw materials for petrochemical processes.

**Scheme 1:**
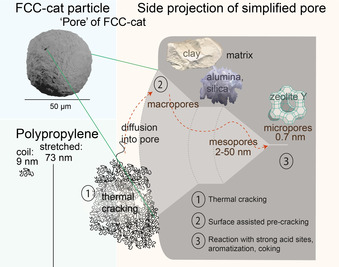
Illustration of the size of the different kinds of pores present in Fluid Catalytic Cracking (FCC) catalysts and the three main components of the catalyst, where zeolite Y mainly gives rise to microporosity and clay and alumina give rise to macro‐ and mesoporosity. Pore sizes are drawn to scale in comparison to the low molecular weight polypropylene polymer (Mw≈12 000 g mol^−1^ and Mn≈5000 g mol^−1^) used in this work.

A lot of research has gone into the design of FCC catalysts, which are spherical particles with a diameter of 50–150 μm that consist of four main components (Scheme 1). The zeolite contains a high density of Brønsted acid sites (BAS) and is most active for cracking and aromatization reactions, but is the least accessible due to their pore size of 7 Å in zeolite Y (FAU topology). Alumina and silica are active in pre‐cracking larger molecules able to access the mesopores of the FCC catalyst particles. They are also acidic, giving rise to Lewis Acid Sites (LAS) in addition to BAS, and thus active for aromatization. Lastly, clay is used as a filler material to give the catalyst particle its round shape and to bind the other components.[^25^, ^29^, ^30^]

During industrial FCC operation, metals, mostly Fe, Ni and V originating from the VGO feedstock and reactor fouling, deposit on the catalyst particles making a large fraction of the zeolite domains inaccessible and causing changes in the overall morphology of the catalyst.[^31^, ^32^, ^33^] In the regenerator of the FCC unit, catalysts are also subjected to steaming leading to dealumination and further deactivation of the zeolite domains.[^34^, ^35^, ^36^] In the FCC process, on average 0.16 kg of catalyst is necessary for the conversion of a barrel of crude oil. During operation the catalyst ages due to metal contamination, steaming and attrition and needs constant replenishment with fresh catalyst. This results in a mixture of catalyst particles with varying degrees of deactivation; this mixture is denoted as equilibrium catalyst (ECAT). When fresh catalyst is added, this ECAT is removed from the processing unit and discarded.^[9]^

This catalyst waste product, however, was shown to convert polyolefins to gasoline‐like products, comparable to what is obtained with fresh catalysts apart from a decrease in the C1‐4 gaseous fraction and a higher olefin content. While this was also observed by other authors, previously a lower BTX content was obtained with ECAT, likely due to a different reactor configuration used.^[37]^ Interestingly, coke deposition was less on the waste catalyst than on a fresh FCC catalyst.[^38^, ^39^, ^40^, ^41^, ^42^] ECAT also showed a higher stability upon several regeneration cycles.^[43]^ This shows the great potential of using this waste catalyst material from the crude oil refining industry to convert plastic waste into aromatics, olefins and paraffins. Questions, however, remain on how plastic and catalyst can best be contacted. In contrast to VGO, plastics are solid at room temperature and very viscous when molten.[^44^, ^45^] In addition, the long polymer chains cannot directly enter the micropores of the catalyst (Scheme 1) and it can be speculated that thermal pre‐cracking plays an important role in the mechanism of polymer conversion over fresh as well as waste FCC particles. These factors and the underlying mechanism of polyolefins conversion over these catalyst materials determine the final product distribution.^[40]^ Thus, a better understanding of those mechanisms will provide handles to achieve a higher value product than gasoline, i.e., a pure stream of methylbenzenes or benzene, toluene and xylene (BTX), used to enhance the octane number of fuels and as feedstock for e.g., the production of fine‐chemicals, commodity goods, plastics and medicine.

In this work, we explored the potential and underlying reaction pathway and transport limitations when using the waste catalyst from an FCC unit, that is, ECAT, to convert PP into mixtures of methylbenzenes and alkanes. To understand the role of the different components of FCC materials, we also investigated pure zeolite Y, a fresh FCC catalyst, an FCC catalyst where zeolite Y has been replaced with clay filler (FCC‐NZ), and a fresh FCC catalyst impregnated with Fe or Ni via incipient wetness impregnation. We focused on the direct catalyst/polymer interaction in particular and investigated the interplay between thermal pre‐cracking, catalytic pre‐cracking and aromatization. To understand the importance of thermal pre‐cracking, we also performed a two‐step reaction, where in the first step PP was cracked without the addition of a catalyst and the thermal cracking oil was then converted together with a catalyst in the second step. Most previous studies of polyolefin conversion over FCC‐cat and ECAT have used fluidized bed reactors, which do not allow the analysis of the catalyst at different stages of the reaction. In this work, all reactions were performed in a semi‐batch reactor using a heating ramp, which allowed online analysis to capture the progression of the reaction by gas chromatography (GC). The reaction products were analyzed by mass spectrometry coupled to a GC (GC‐MS) and a GC with a flame ionization detector (GC‐FID). To visualize the location of aromatic products and carbonaceous deposits in the pores of the catalyst with progression of the reaction, we have quenched the reaction at relevant time points and characterized the polymer/catalyst mixtures using confocal fluorescence microscopy (CFM). Infrared spectroscopy (IR) was used to characterize the C−H stretching of partially cracked PP early on in the reaction. The degree of ordering or graphitization of carbonaceous deposits (coke) on catalysts recovered after completion of the reaction were characterized using Raman spectroscopy. Finally, thermogravimetric analysis (TGA) and Ar physisorption were used to determine the quantity of deposited coke and the decrease of the pore volume due to pore blockage, respectively.

## Results and Discussion

The main characteristics that determine the catalytic behavior of the three catalysts, FCC‐cat, FCC‐NZ, and ECAT, are acidity, pore volume distribution, and metal content. All three catalysts were extensively investigated in previous work of our group.[^29^, ^31^, ^32^, ^33^, ^34^, ^35^, ^36^, ^46^, ^47^, ^48^, ^49^, ^50^, ^51^, ^52^, ^53^] We compared the acidity of the three catalysts using pyridine FT‐IR spectroscopy (Figure S8). The small amount of BAS observed on FCC‐NZ stems from the silica‐alumina of the matrix, while the much higher BAS density on FCC‐cat is associated with the presence of zeolite Y. The amount of BAS on ECAT was as low as on FCC‐NZ, indicating that the zeolite domains are largely inaccessible due to metal deposition and deactivated due to steaming.[^32^, ^35^, ^36^] This is also supported by the Ar physisorption results, which show a micropore volume for ECAT (49.4 μL g^−1^), just over half of that compared to FCC‐cat (84.0 μL g^−1^). FCC‐NZ exhibited the smallest micropore volume (3.9 μL g^−1^) due to the absence of zeolite, while the mesopore volume was found to be comparable for all three catalysts (ECAT: 88.7 μL g^−1^; FCC‐cat: 70.3 μL g^−1^; and FCC‐NZ: 82.4 μL g^−1^) (Table S6, Figure S9).

**Figure 1:**
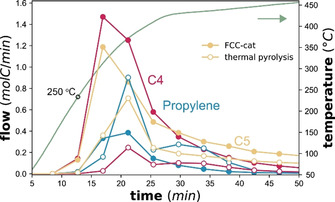
Molar flow of C3−5 products normalized by carbon number, measured over time of reaction and temperature in the reactor (heating rate 20 °C min^−1^) for reaction of 2.5 g Polypropylene (PP) without a catalyst (open symbols) and with 1.25 g of FCC‐cat (*closed symbols*). See Figure S10 for the product evolution over the FCC‐NZ and ECAT catalyst materials.

**Figure 2:**
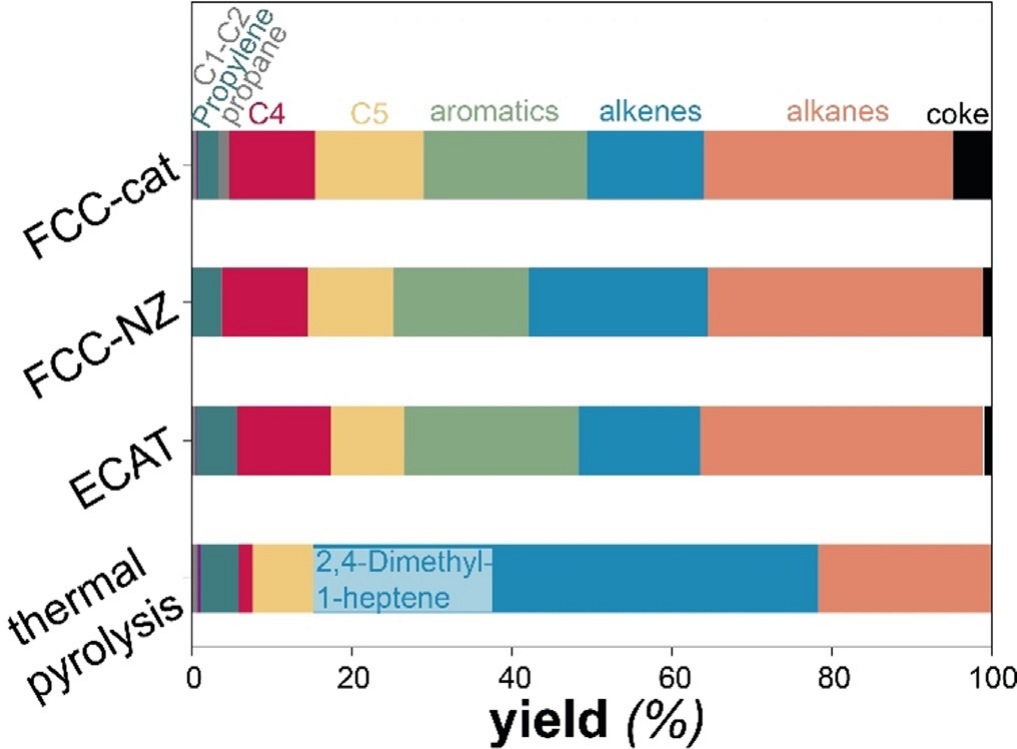
Yields of products obtained from reaction at standard reaction conditions (Supplementary Information). C1−5 products were integrated from online GC analysis (Figure 1) and >C5 products were identified and classified offline via GC‐MS and quantified via GC‐FID (Figure S10). Coke amounts were determined by TGA of the spent catalysts (Figure S12).

Catalyst testing was performed by loading PP, and, if applied, the catalyst, into the autoclave reactor (Figure S1) and applying a heating ramp of 20 °C min^−1^ up to ≈450 °C. C1‐C5 products were continuously measured by online GC. To test, whether the size of the PP pellets influences the reaction, an experiment was performed in which the PP pellets were previously crushed. This only lead to very small changes in the product evolution (Figure S17). Full conversion was reached in all cases after 45 min. Generally, the presence of a catalyst lowered the onset temperature for product evolution by about 100 °C to 250 °C and caused an increase in the formation of C4‐5 hydrocarbons, which was the highest for FCC‐cat as also observed by other authors (Figure 1, Figure S10).^[37]^ While C1‐C5 hydrocarbons were analyzed by online GC, the heavier condensable products were collected during reaction and analyzed afterwards by offline GC‐MS and GC‐FID. Adding a catalyst to the reactor caused a dramatic increase in aromatic yields, mostly methylbenzenes in the condensable product (>C5), which were not produced without a catalyst (Figure 2, Figure S11). The same type of products was formed with all three catalyst materials. The fact that significant amounts of aromatics was produced over FCC‐NZ demonstrates the aromatization activity of the matrix. The higher aromatization activity of FCC‐cat is explained by the presence of the zeolite. However, despite the inaccessibility of the zeolite on ECAT and thus the low BAS, the aromatics content was not lower than from FCC‐cat (Figure 2).

Aromatization in the zeolite pores predominantly proceeds via hydrogen transfer to an alkene to form an alkane.^[54]^ In the ECAT, however, the zeolite pores are largely inaccessible (Figure S8) and aromatization can be assumed to mainly proceed via metal‐assisted dehydroaromatization in the matrix forming molecular hydrogen^[25]^ and to a lesser extent via hydrogen transfer to an alkene. This is evident from the higher H_2_ production (Figure S12). The zeolite is not necessary for aromatization of PP as FCC‐NZ also formed aromatics and ECAT even produced the same amounts of aromatics as FCC‐cat. Interestingly, the discarded ECAT caused the least coke deposition (1.98 wt. %) compared to FCC‐cat (9.02 wt. %) and FCC‐NZ (2.19 wt. %) (Figure 2, Figure S13). A lower amount of carbonaceous deposits on ECAT than on FCC‐cat was also observed during catalytic pyrolysis of HDPE.^[38]^ To test whether the presence of metals alone can lead to this improved performance, we have tested FCC‐cat impregnated with either Ni or Fe. While slightly improving aromatics yields, this was accompanied by higher coke amounts than on FCC‐cat (Figure S14,15). This suggests that the presence of the zeolite component is detrimental to catalyst lifetime when processing PP and that only the combination of the presence of metals and the absence of strong and confined BAS leads to an improved performance of ECAT and highlights the potential of using this waste catalyst for conversion of polyolefins.

Figure 1 shows that the interaction of PP with the catalyst surface of the catalyst allows for cracking at lower temperatures than thermal pyrolysis. This was observed previously and it was hypothesized that this interaction is somehow limited to the outer external surface of the catalyst particle.^[41]^ To understand this better, partially cracked PP and catalyst were recovered after 13 min of reaction when the first products started to form at approximately 250 °C. At this temperature, the activation energy barrier for thermal cracking cannot be overcome yet. The recovered samples were analyzed with FT‐IR in two different modes to compare possible cracking in the bulk of the PP to cracking at the catalyst surface.

**Figure 3:**
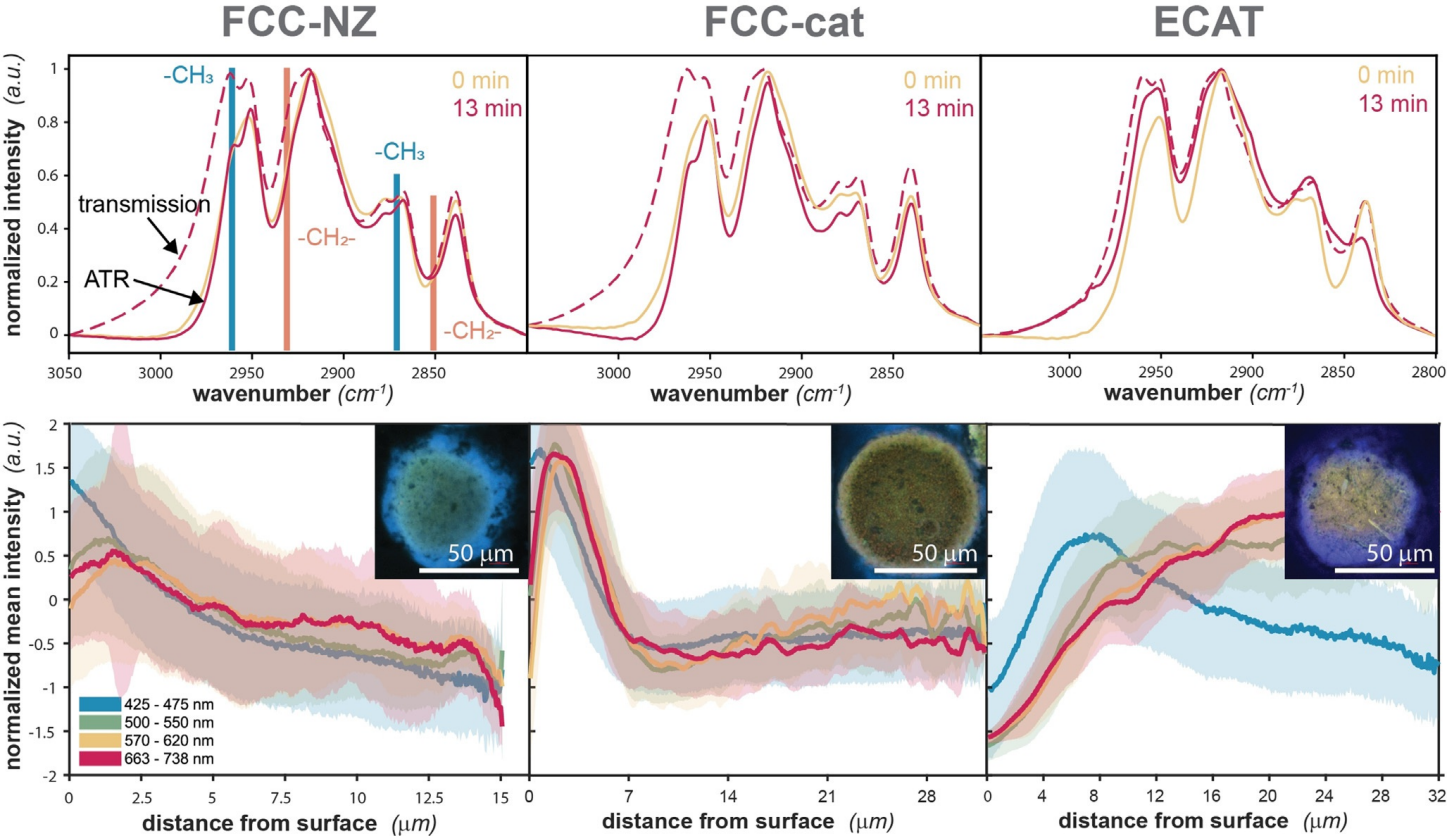
*Top*: FT‐IR spectra of PP/catalyst mixtures after quenching the reaction at 250 °C (all catalysts, red) and 300 °C (only ECAT, plum). Spectra measured in transmission (dashed) mode show a shoulder in the region associated with olefinic C−H stretching vibrations compared to spectra measure in ATR mode (solid). A spectrum of pure PP is indicated in yellow. *Bottom*: Radial intensity profiles of CFM images of microtomy cross‐sectioned FCC‐NZ, FCC‐cat and ECAT particles after 13 min of reaction. The error bars correspond to the standard deviation of intensity of the fluorescence. For profiles of all imaged particles rf. Figure S20.

Attenuated total reflectance (ATR)‐IR spectroscopy is sensitive to the material directly in contact with the ATR crystal, having a penetration depth of 0.64–0.7 μm in the wavenumber region of interest (ref. “Infrared and Raman spectroscopy” in the supplementary information). The microscopy image of a cut‐through of the catalyst/polymer mixture (Figure S16) shows that the particles were surrounded by a polymer layer thicker than 0.7 μm. Thus, the ATR mode can be used to characterize the degree of cracking of PP surrounding the catalysts. In transmission mode, on the other hand, the absorption of the entire PP/catalyst mixture is measured, and the products formed on the catalyst are also captured. For FCC‐cat and FCC‐NZ, the PP material far away from the catalyst surface only showed slight signs of cracking (Figure 3). In contrast, the transmission FT‐IR data displays an intense shoulder associated with olefinic C−H stretching and a more intense band associated with the C−H stretching of methyl‐groups (2962 cm^−1^)^[55]^ for all catalysts under study. This suggests that surface‐assisted cracking in the catalyst had already progressed further than in the bulk of the plastic. However, for ECAT, even in ATR mode a more intense olefinic C−H stretching region was observed, suggesting that cracking was faster on this catalyst and the products had already started to diffuse out through the plastic layer surrounding the catalyst material.

To visualize the aromatization inside the catalyst particle, CFM was used. CFM excites and detects the fluorescence of aromatics,^[56]^ while PP, alkanes and alkenes do not fluoresce in the wavenumber regions used.^[57]^ This technique was previously used to study the accessibility and strength of acid sites on FCC‐cat,[^36^, ^46^, ^48^] ECAT,^[52]^ clay‐bound ZSM‐5‐based catalyst bodies^[58]^ and different types of pure zeolites[^59^, ^60^, ^61^, ^62^, ^63^] using fluorescent probes and studying reactions *operando*. To record a high‐resolution image of the inside of the catalyst particle, we have mapped microtomy cuts (Figure S6). The partially cracked PP, as identified in the microscopy image (Figure S16) and by FT‐IR spectroscopy showed no significant fluorescence (Figure 3, Figure S20). However, a high fluorescence signal was observed inside the catalyst particle. A higher intensity of fluorescence was also observed in the outer ring of the catalyst particle for FCC‐cat and FCC‐NZ, suggesting that the reaction had not progressed into the center of the catalyst particle (Figure 3). For ECAT, the radial intensity profile shows an increase towards the center of the particle. This difference in the intensity profiles for FCC‐NZ and FCC‐cat compared to ECAT can be explained by assuming that the uncracked polymer cannot enter deeper into the pore network of the catalyst and that pre‐cracking precedes diffusion.^[64]^

The PP that was used in these experiments has a rather low molecular weight (M_w_≈12 000 g mol^−1^), the root‐mean‐square end‐to‐end distance in its coiled state is about 9 nm and the average length of the fully extended polymer 73 nm (Section S8 for calculations). But even with these relatively small polymers, the chains are suspected not to be able to enter deep into the catalyst pores without pre‐cracking, especially because they first have to untangle. As revealed by FT‐IR spectroscopy, the ECAT material exhibited a higher pre‐cracking activity, likely due to Fe, Ni and V deposited there, and thus diffusion into the pores of the catalyst was enhanced and aromatization there facilitated. This leads to the higher fluorescence signal in the center of ECAT particles (Figure 3).

**Figure 4:**
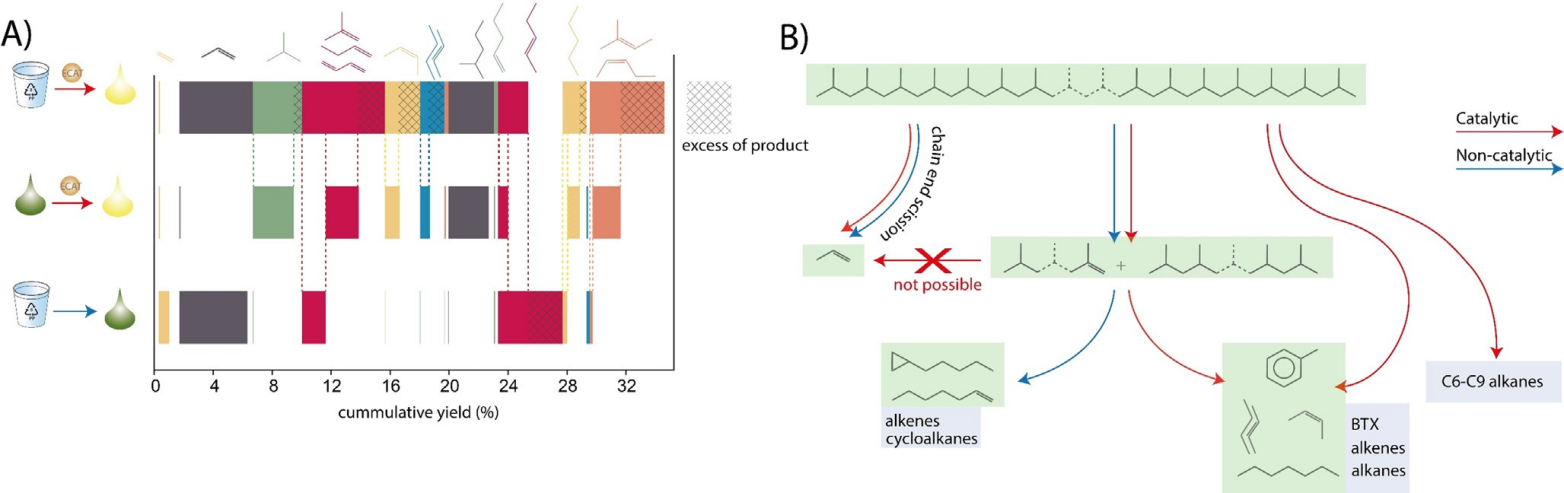
*Panel A*: cumulative yields of C1‐5 products formed during purely thermal pyrolysis (*bottom*), catalytic pyrolysis of the pyrolysis oil using the ECAT catalyst (*middle*) and direct catalytic pyrolysis of polypropylene (PP) using the ECAT catalyst (*top*). The bars of each product are shifted to align with the same product produced in the other processes to compare their quantities in the different runs directly. *Panel B*: various pathways to products through catalytic and non‐catalytic one and two‐step reactions. This Scheme was developed based on the findings reported in Panel A.

CFM of the recovered polymer/catalyst mixtures (Figure 3) revealed transport limitations to the polymer/catalyst interaction and it is interesting to determine the extent to which the polymer cracks thermally before interacting with the catalyst. We therefore first conducted the reaction non‐catalytically and then converted the obtained condensable product again using ECAT. If the products obtained from this two‐step conversion and the products obtained from directly contacting PP with the catalyst were found to be identical, complete thermal cracking can be assumed to precede any interaction with the catalyst. It was found that the same compounds were produced in the two‐step process as from direct catalytic cracking and were markedly different from the products obtained with thermal pyrolysis (Figure 4). The product amounts, however, were not the same. When directly reacting PP and ECAT, long PP chains first have to be cracked into smaller molecules at the outer surface and in the pore mouths of the macropores before they can diffuse into the pore network of the catalyst and aromatize. Some initial cracking products directly leave the catalyst surface before being aromatized.

This is evident from the fact that some products of direct PP conversion over ECAT were not formed at all in the two‐step process. The most remarkable difference is the absence of propylene. Propylene is believed to mainly form via chain‐end scission^[65]^ and our results suggest that it forms from long‐chain polymers not present anymore after thermal pyrolysis. Propylene also formed earlier when a catalyst surface was provided and in higher amounts when more catalyst was added or when ECAT was crushed prior to reaction (Figure S18). Alkenes below C9 were formed in much lower amounts from pyrolysis oil than from PP and are thus also associated with direct PP/catalyst interaction (Figure S19). Remarkably, the aromatics content was increased by a factor of 1.4 (Figure S19). Likely, the smaller alkenes and alkanes formed during thermal cracking can more easily enter deep into the complex pore network of the catalyst and thus aromatize faster.

The ability of the matrix to form aromatics was demonstrated by the significant aromatics content obtained with FCC‐NZ. In the first 13 min of reaction, the aromatization in the matrix also dominated for FCC‐cat. This is clear from the fact that the condensable products formed up to this point were almost identical for FCC‐cat and FCC‐NZ (Figure S21). More aromatics were formed later in the reaction over FCC‐cat, while FCC‐NZ ceased to produce more aromatics. The late formation of aromatics in the zeolite domains again suggests that this process is limited by pre‐cracking and diffusion. This is further supported by the fact that even more aromatics were formed when converting PP over zeolite Y directly or over a mixture of 40 wt. % zeolite Y mixed with FCC‐NZ, which corresponds approximately to the weight distribution in FCC‐cat (Figure S22). In this case, zeolite Y is directly accessible.

**Figure 5:**
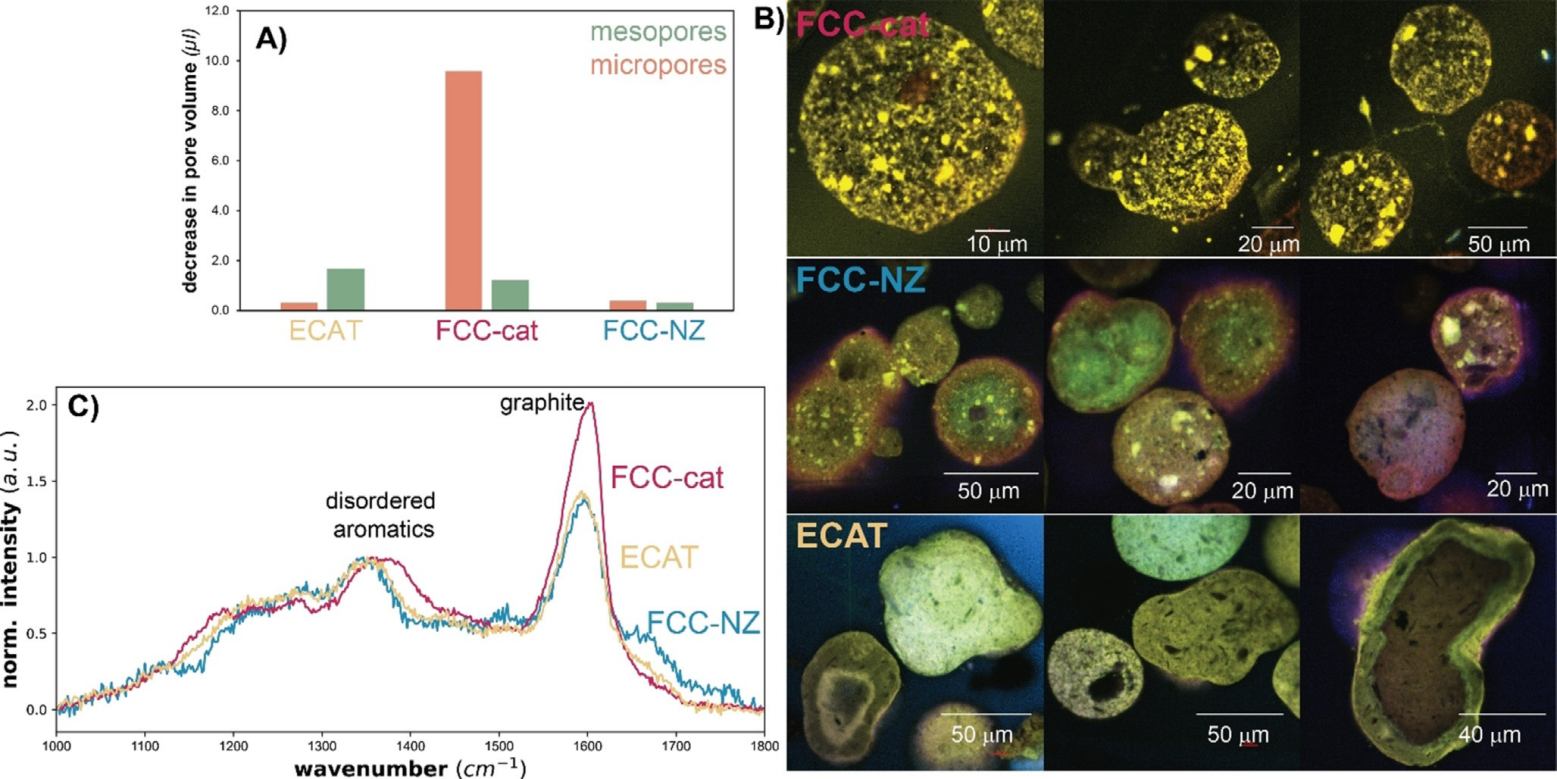
*Panel A*: Decrease in micro‐ and mesopore volume calculated by comparing Ar physisorption results before and after reaction. *Panel B*: Confocal fluorescence microscopy (CFM) images of microtomy cross‐sections of FCC‐cat, FCC‐NZ and ECAT. Fluorescence Intensities of the CFM images are scaled for optimal brightness and visibility *Panel C*: Raman spectra recorded on the spent catalyst materials under study.

Aromatics are precursors to coking. When they grow larger in size in the meso‐ and macropores of the matrix they can still leave the catalyst, but when they form in the micropores of the zeolite they accumulate in the confined space.[^66^, ^67^, ^68^] This leads to an extensive micropore blockage for FCC‐cat as was observed with Ar physisorption performed on spent FCC‐cat (Figure 5, *panel A*). The distinct bright spots in the CFM image taken after full reaction correspond to the zeolite domains for FCC‐cat and are not visible for ECAT, which again suggests that the zeolite domains are inaccessible on ECAT (Figure 5, *panel B*). Some bright spots appear on FCC‐NZ but much bigger in size, suggesting that these are silica‐alumina domains. The fact that the distinctive bright features on FCC‐cat only appeared after full reaction, further confirms that matrix pre‐cracking precedes aromatization in the zeolite domains. It is noted that total fluorescence cannot be used as a measurement for quantity of coke formed as the nature of coke influences the fluorescence. Carbonaceous deposits formed on FCC‐cat exhibit a more graphitic nature as shown by the relatively higher intensity of the G band in the Raman spectrum compared to the D band (Figure 5, *panel C*).

## Conclusion

A waste refinery catalyst, namely an equilibrium Fluid Catalytic Cracking (FCC) catalyst (further denoted as ECAT), shows great potential for the conversion of polypropylene (PP). The metals, including Fe, Ni, and V deposited on the ECAT catalyst during FCC operation, have a favorable effect as they enhance the aromatization and pre‐cracking activity of the catalyst matrix. Zeolite domains on ECAT are blocked by metals and deactivated due to steaming in the regenerator of the FCC unit, which leads to a decreased coke deposition. This demonstrates that the strong acidity of the zeolite material and the related micropore structure are not necessary for aromatization of PP and even detrimental regarding the lifetime of the catalyst for processing PP. Furthermore, it has been shown that Confocal Fluorescence Microscopy (CFM) is a powerful technique in determining the extent of catalyst particle utilization and the location of the coke deposition. The aromatization is limited by pre‐cracking in the matrix, because the uncracked PP chains cannot diffuse into the pore channels of the catalyst. This pre‐cracking is also enhanced over the ECAT material. The aromatics content can be increased further when the reaction is conducted in two steps, where PP is first thermally pre‐cracked and the resulting product is contacted with the catalyst, because transport is enhanced. The evolution of products at low temperatures is largely due to the direct interaction of PP with the outer surface of the catalyst particle and not due to radical reactions in the bulk of the plastic. Thus, to achieve lower energy requirements for the catalytic conversion process, it is beneficial to increase the polymer/catalyst contact area as much as possible.

## Conflict of interest

The authors declare no conflict of interest.

## Supporting information

As a service to our authors and readers, this journal provides supporting information supplied by the authors. Such materials are peer reviewed and may be re‐organized for online delivery, but are not copy‐edited or typeset. Technical support issues arising from supporting information (other than missing files) should be addressed to the authors.

Supplementary
