# Supplementary material for: Plastic Waste Conversion over a Refinery Waste Catalyst
Source: Angew Chem Int Ed Engl. 2021 Jun 15;60(29):16101–8. doi: 10.1002/anie.202104110 (PMC8362022; doi:10.1002/anie.202104110)
Supplement: Supplementary file 1 — Supplementary [file ANIE-60-16101-s001.pdf]

Angewandte Chemie
Eine Zeitschrift der Gesellschaft Deutscher Chemiker

Supporting Information

Plastic Waste Conversion over a Refinery Waste Catalyst

*Ina Vollmer[+], Michael J. F. Jenks[+], Rafael Mayorga González, Florian Meirer, and Bert M. Weckhuysen**

anie_202104110_sm_miscellaneous_information.pdf

Contents

Experimental Procedure 2

Supplementary Results and Discussion 12

S1 Analysis of scattering effects in confocal fluorescence microscopy z-stack images 12

S2 Pyridine FT-IR 13

S3 Ar physisorption results 13

S4 Product evolution 14

S5 Analysis of condensable products 14

S6 Hydrogen evolution profiles 15

S7 Thermogravimetric analysis with online mass spectrometry 16

S8 Calculation of polymer chain length 17

S9 Influence of polymer pellet size 18

S10 Two-step reaction 19

S11 Quenching the reaction after 13 min 20

S12 Effect of zeolite Y 21

S13 References 22

Experimental Procedure

Materials

Isotactic polypropylene (PP) pellets with a 2-8 mm diameter, average M_w ~ 12,000 g/mol and average M_n ~ 5,000 g/mol was obtained from Sigma Aldrich (428116) and used as received. A fresh FCC catalyst (FCC-cat, BET surface area: 261.1 m_2/g) and an FCC catalyst without zeolite (FCC-NZ, BET surface area: 83.6 m^2/g) as well as an equilibrium catalyst (ECAT, BET surface area: 183.2 m^2/g) was obtained from Albemarle. The ECAT material was calcined prior to use, heating to 500 °C at 5 °C/min with a hold time of 2 h at 120 °C and 5 h at 500 °C. Furthermore, a zeolite Y material with a Si/Al ratio of 15 was obtained from Zeolyst (CBV720, BET surface area: 872.9 m^2/g) in its proton form and used as received.

Physisorption Measurements

Ar adsorption was performed on a TriStar II 3020 Version 3.02 (Micromeritics) at liquid nitrogen temperature for determination of the BET surface area. The t-plot method was used to determine the micropore volume between a thickness of 0.35 and 0.5 nm using the Harkins and Jura equation to calculate the thickness ($0.1[\frac{13.99}{0.034-\log(\frac{p}{p_0})}]$). The total pore volume was calculated from the maximum volume adsorbed (p/p0 = 0.732 for FCC-cat and 0.995 for all other samples) during Ar adsorption measurements multiplied by the density conversion coefficient (0.00128), which accounts for the condensation of Ar upon adsorption in the micropores to a liquid-like state. The mesopore volume was calculated as the difference between total pore volume and micropore volume. Before adsorption, the catalyst materials were dried under a flow of N_2 at 250 °C for 16 h. FCC-cat was dried under dynamic vacuum at the same temperature.

Catalytic Pyrolysis

In a typical pyrolysis experiment, 2.5 g of polypropylene (PP) was loaded together with 1.25 g of catalyst yielding a PP:catalyst ratio of 2:1 into a 50 ml Parr autoclave reactor equipped with a simple stirrer. The stirring speed was 100 rpm. A 50 ml/min flow of N_2 was sent through the reactor and served as carrier gas for online gas chromatographic (GC) analysis of C1-C5 products formed. The remaining products are collected in an ice-cooled condenser (**Figure S1**). The gas lines upstream of the reactor and between reactor and condenser were kept at 300 °C.

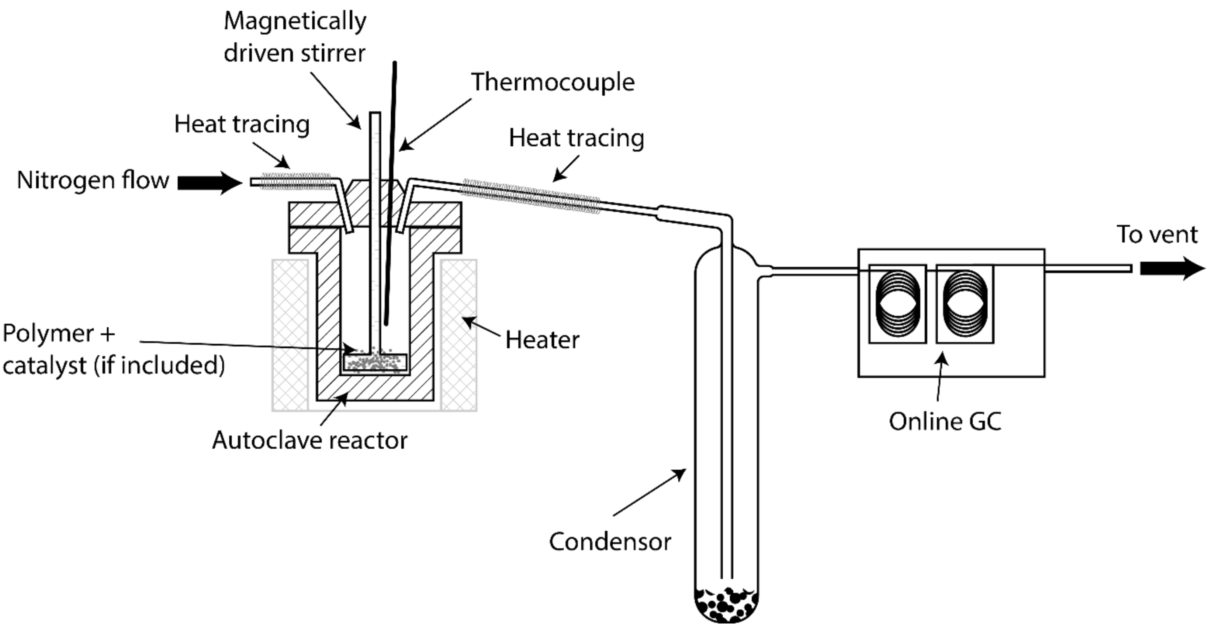

Figure S1. Schematic of the reactor setup used in the catalytic experiments.

The reactor was heated up to 420 °C using a power profile, which was previously calibrated to yield a 20 °C/min heating ramp, when the reactor is filled with silicon carbide powder (Alfa Aesar, 46 grit) (**Figure S2**). The temperature ramp was not left to be controlled by the internal thermocouple, as the temperature measured depends on the filled volume and the thermal conductivity of the catalyst material and we deemed keeping the power input the same a more fair comparison.

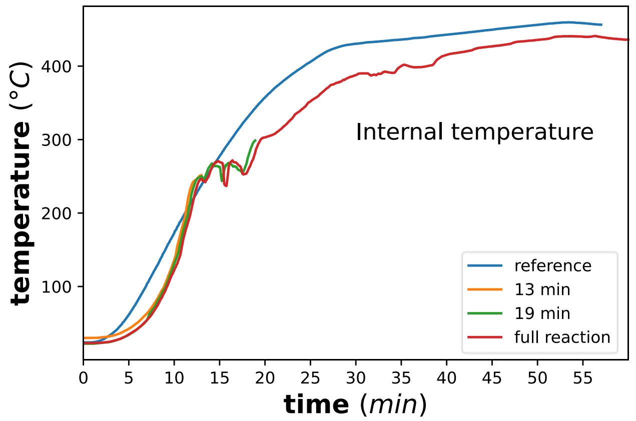

Figure S2. Temperature measured inside the during reaction of PP with ECAT1 for 13 and 19 minutes reaction time as well as for full reaction under standard conditions (rf. Section 2.4 in main text). During reaction, the thermocouple is placed above the catalyst/PP mixture as it would otherwise interfere with the stirrer rotation (Figure S1). The reference temperature was measured in an experiment, where no reaction was taking place and the catalyst/PP mixture was replaced by 13.4 g of SiC. No stirring was performed and a longer thermocouple employed to measure the temperature inside the SiC.

Reactions were performed at near ambient pressure of about 1.1 atm. A slight increase in pressure is observed due to pressure buildup in the injection loops of the GC, but is consistent from experiment to experiment. The N_2 carrier gas flow was used as internal standard to correct for the flow volume increase as products form. Online GC analysis is performed using an Interscience CompactGC equipped with a TCD coupled to a 2 m SC-ST column of 1/16 in diameter and a 0.5 m Hayesep Q pre-column with 1/16 in diameter to detect H_2 and N_2 formation. C1-5 hydrocarbons were detected on an FID with 10 m RT-Qbond column of 0.32 mm diameter. To quench the reaction, the heating oven

mantle was switched off and moved away from the reactor, which was subsequently immersed in an ice bath. Catalysts and carbonaceous deposits or remaining plastic were collected after reaction for further analysis. The two-step experiment was essentially performed the same way, except that another GC was employed and the condenser was kept at room temperature. For the two-step experiment, an Interscience CompactGC was used, equipped with a TCD coupled to a Molsieve 5A (10 m*0.53 mm) and a Rt-Qbond (3 m*0.32 mm) pre-column to detect H_2 and N_2 formation. C1-3 hydrocarbons were detected on an FID with aAl2O3/MAPO (15 m*0.32 mm) and an Rtx-1 (5u, 3 m*0.32 mm) precolumn, while C4-5 hydrocarbons were analyzed on an FID with an RTx-1 (3u, 15 m*0.32 mm) and an Rtx-1 (3u, 3 m*0.32 mm) precolumn.

Product evolution is presented as mol of carbon per minute, which was obtained using the formulas below. Since the molar flow of N_2, F_{N_2} was constant, diving this flow by the molar concentration of N_2 at each injection, i provides the total molar flow exiting the condenser. In this way the N_2 flow was used as an internal standard to account for the change in volumetric flow due to product evolution.

$$F_{total,i} = \frac{F_{N_2}}{y_{N_2,i}}, F_{N_2} = const. = 50\frac{ml}{min}\ or\ 50ml * \frac{10^5 Pa}{298\ K * 8.314\frac{J}{mol * K}} = 2.018\ mmol/min$$

The molar concentration of N_2 at each injection was determined by calibration of the peak area of N_2 using the first three injections prior to starting the reaction and after the flow had stabilized.

$$y_{N_2,i} = \frac{\frac{\sum_{i=-3}^{i=0} A_{N_2,i}}{3}}{A_{N_2,i}} * 100\%$$

The carbon molar flow of each hydrocarbon was calculated by multiplying by carbon number x.

$$F_{C_xH_y,i} = y_{C_xH_y,i} * F_{total} * x$$

The concentration of each C1-5 hydrocarbon was calculated by dividing the peak area of that hydrocarbon by a calibration factor determined through injecting a known gas mixture, $CF_{C_xH_y,i}$.

$y_{C_xH_y,i} = \frac{A_{C_xH_y,i}}{CF_{C_xH_y,i}}$, where $CF_{C_xH_y,i} = CF_C * x$ with CF_C being the calibration normalized by carbon number. On a FID the response is proportional to the carbon number within a small error margin.

The cumulative yield of each C1-C5 hydrocarbon was calculated through integration of the molar flow of C1-5 hydrocarbons over time and multiplying by their molecular weight $M_{C_xH_y}$.

$$Y_{C_xH_y}[g] = \frac{M_{C_xH_y}}{x} * \int_0^{t_{final}} F_{C_xH_y,i} dt$$

Coke yields were determined from total mass loss measured during Thermogravimetric Analysis (TGA) after the initial water desorption step, assumed to be completed after reaching a temperature of 300 °C. Where $w_{catalyst}$ was typically 1.25 g.

$$Y_{coke}[g] = (m_{300\,℃} - m_{end})/m_{300\,℃} * (1 + (m_{300\,℃} - m_{end})/m_{300\,℃}) * w_{catalyst}$$

The yield of condensable products was then calculated via the mass balance and via weighing the condenser before and after reaction. However, determination via the mass balance was deemed more accurate.

$$Y_{liquid} = m_{PP} - Y_{coke} - \sum Y_{C_xH_y}$$

The calibration factor CF_C for the FID of the online GC used for analysis of gaseous compounds (C1-5) for experiments presented in **Figure 1-2** were determined from the calibration injections shown in **Table S1** for methane and ethane. The calibration gas was obtained from Linde Gas Benelux B.V. and contained 20.30mol% methane, 5.04 mol% ethane, 2.02mol% ethylene, 10.00mol%

carbon monoxide, 20.00mol% carbon dioxide and 20.00mol% hydrogen, the rest being nitrogen. Ethylene was disregarded in the calibration as it is not very stable upon longer storage.

Table S1. Peak areas obtained from integration of the chromatograms of the calibration injections with statistical analysis and calibration factors obtained.

chromatogram #	Concentration (mol%)		Areas (mV*min)	
	Methane	Ethane	Methane	Ethane
1	20.30	5.04	12.2519	6.5576
2	20.30	5.04	12.2437	6.6004
3	20.30	5.04	12.192	6.5475
average			12.2292	6.5685
std deviation (%)			0.27%	0.43%
Calibration factor (mVmin/mol%)			0.602424	1.303274

The calibration factor per carbon is determined by interpolation of the calibration factors in **Table S1** (**Figure S3**).

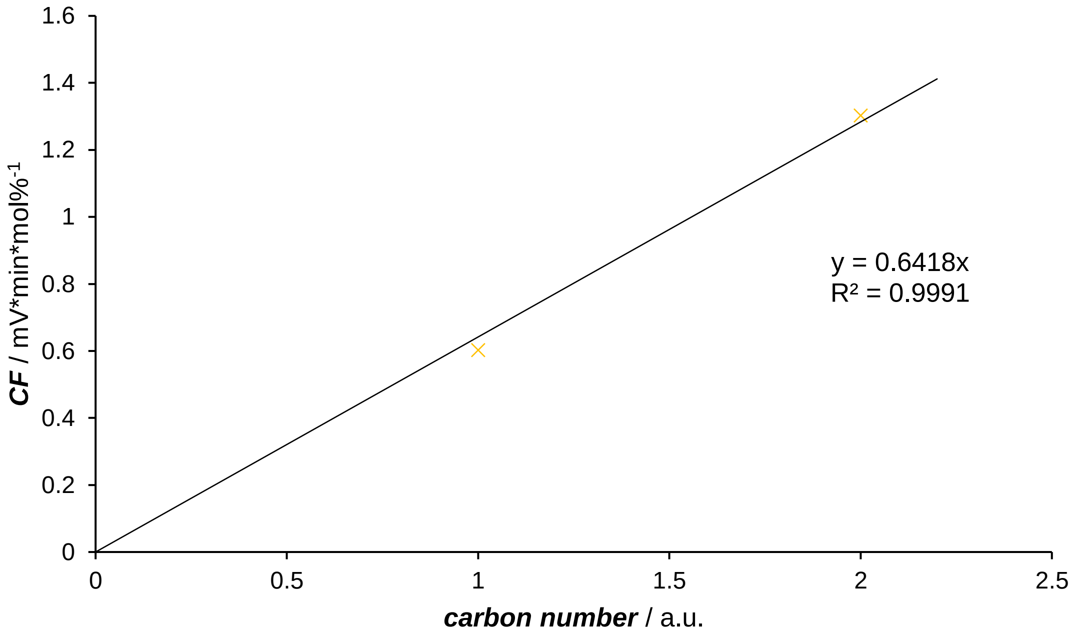

Figure S3. Interpolation of the calibration factors for methane and ethane (Table S1) to obtain a calibration factor per carbon number used to analyze the gaseous products presented in Figures 1-2.

Calibration for the two-step experiment (**Figure 4**) was performed with a more complicated gas mixture (Linde Gas Benelux B.V.) to allow for more detailed analysis (**Table S2**).

Table S2. Composition of the calibration gas mixture used for calibration for analysis of the two-step reactions (Figure 4).

**compound name**	**Concentration (mol%)**
cis-2-pentene	0.026
2-methyl-2-butene	0.034
trans-2-pentene	0.041
pentane	0.047
2-methyl-1-butene	0.051
trans-2-butene	0.056
2,2-dimethylpropane	0.071
pent-1-ene	0.082
3-methyl-1-butene	0.099
Isopentane	0.112
propyne	0.139
isobutene	0.161
1,3-butadiene	0.147
cis-2-butene	0.233
acetylene	0.229
butane	0.239
propane	0.25
1-butene	0.272
isobutane	0.413
propylene	0.806
ethylene	0.995
ethane	1.69
Ar	4.94
CO_2	5.03
Methane	4.8
CO	9.69
He	10.8

The first FID was used to detect all C1-3 products. Compounds were injected twice per chromatogram:

Table S3. Peak areas obtained from integration of the chromatograms of the calibration injections with statistical analysis and calibration factors obtained.

	Area (pA*min)									
	Methane		Ethane		Ethene		Propane		Propene	
injection	1st	2nd	1st	2nd	1st	2nd	1st	2nd	1st	2nd
carbon number	1	1	2	2	2	2	3	3	3	3
chromatogram #										
1	7.104	7.209	5.009	5.358	2.820	2.871	1.051	1.101	3.187	3.343
2	7.131	7.211	5.031	5.340	2.831	2.868	1.107	1.134	3.374	3.451
3	7.093	7.187	5.009	5.307	2.819	2.857	1.123	1.285	3.413	3.468
average of selected injections	7.109	7.202	5.016	5.335	2.823	2.865	1.094	1.173	3.325	3.421
std deviation (%)	0.28%	0.18%	0.25%	0.49%	0.25%	0.25%	3.44%	8.35%	3.64%	1.98%
concentration (mol%)	4.800	4.800	1.690	1.690	0.995	0.995	0.250	0.250	0.806	0.806
calibration factor	1.481	1.5	2.968	3.157	2.838	2.88	4.375	4.693	4.125	4.244

The calibration factor per carbon is determined by interpolation with carbon number (**Figure S4**).

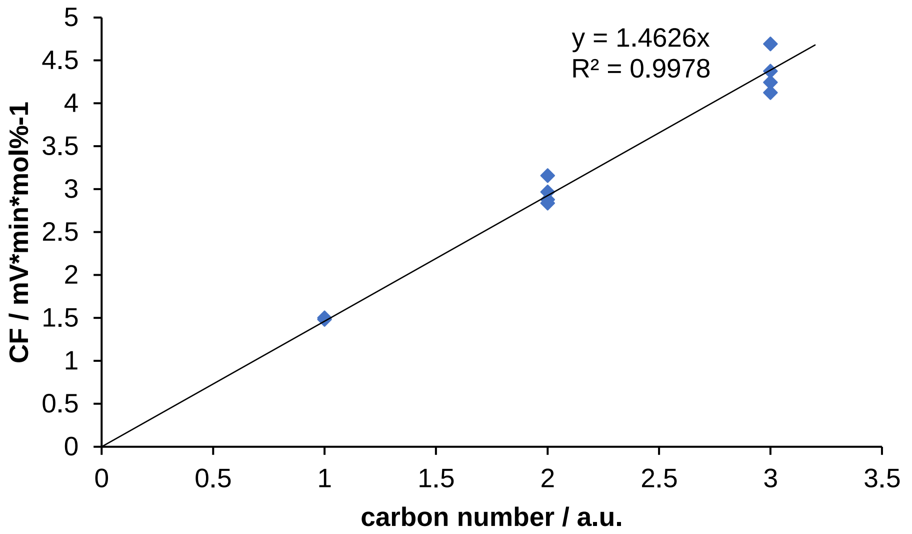

Figure S4. Interpolation of the calibration factors for C1-3 compounds (Table S3) to obtain a calibration factor per carbon number for the first FID used to analyze the gaseous products presented in Figure 4.

The second FID was used to detect all C4-5 products (**Table S4-5**).

Table S4. Peak areas obtained from integration of the chromatograms of the calibration injections on the second FID for C4 compounds with statistical analysis and calibration factors obtained.

chromatogram #	isobutane	isobutene&but-1-ene&1,3-butadiene	cis-2-butene	butane&trans-2-butene	sum C4
1	0.318	2.527	0.009	0.282	3.135
2	0.335	2.684	0.009	0.297	3.325
3	0.339	1.975	0.854	0.312	3.480
average (pA*min)	0.331	2.395	0.290	0.297	3.314
Std. deviation (%)	3.38%	15.54%	168.04%	4.97%	5.21%
concentration (mol%)					1.52
calibration factor (pA*min/mol%)					2.18

Table S5. Peak areas obtained from integration of the chromatograms of the calibration injections on the second FID for C5 compounds with statistical analysis and calibration factors obtained.

	Areas (pA*min)							
chromatogram #	3-methyl-1-butene	isopentane	pent-1-ene	trans-2-pentene	n-Pentane_FID2	2,2-dimethylpropane	cis-2-pentene &2-methyl-2-butene	sum C5
1	0.325	0.355	0.300	0.219	0.130	0.076	0.143	1.548
2	0.341	0.371	0.345	0.186	0.136	0.073	0.144	1.595
3	0.357	0.388	0.317	0.244	0.136	0.081	0.147	1.671
average (pA*min)	0.341	0.371	0.321	0.216	0.134	0.076	0.145	1.605
Std. deviation (%)	4.75%	4.42%	7.09%	13.50%	2.49%	5.37%	1.65%	3.85%
concentration (mol%)								0.56
calibration factor (pA*min/mol%)								2.85

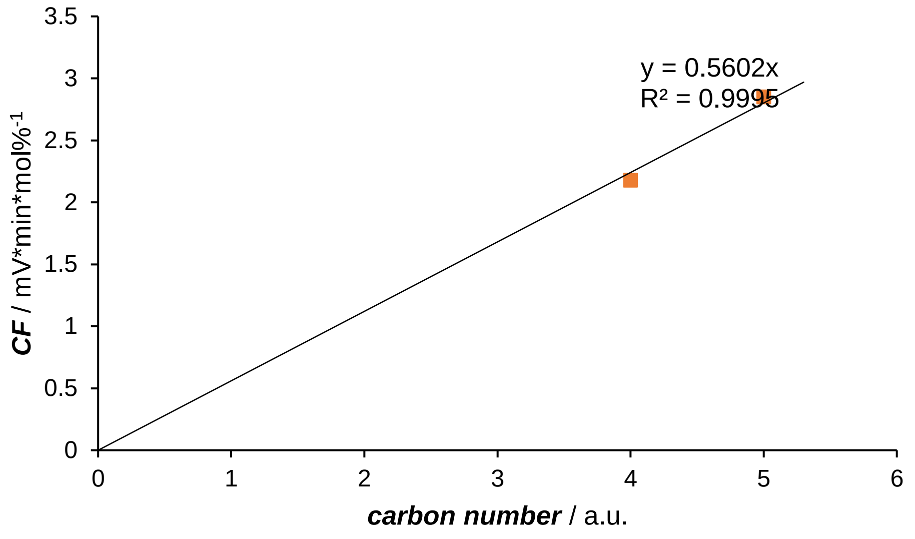

Figure S5. Interpolation of the calibration factors for C4-5 compounds (Table S4-5) to obtain a calibration factor per carbon number for the second FID used to analyze the gaseous products presented in Figures 4.

Analysis of Condensable Products

Gas Chromatography combined with Mass Spectrometry (GC-MS) of the condensed liquids was performed on a Shimadzu GC-2010 instrument equipped with an inert 5% phenylmethyl polysiloxane Agilent VF-5ms column and a Shimadzu GCMS-QP2010 MS. The condensed oil was dissolved and diluted to a 1:100 ratio with dichloromethane. The split ratio was set to 100, with an injection volume of 10 µl at a temperature of 265 °C. The GC data were normalized by its area integrated between 4-15 min retention time. The reaction products were assigned using the Shimadzu GCMS Postrun Analysis software searching the NIST/EPA/NIH Mass Spectral Database (NIST 11). For the noncatalytic pyrolysis run, for most peaks no library match could be found and therefore a custom code was written in python to classify the reaction products as alkanes or alkenes.

The MS fragmentation patterns of alkanes are typically characterized by consecutive peaks corresponding to C_nH_{2n+1} (m/z 29, 43, 57, 71, …), while the C_nH_{2n-1} (m/z 27, 41, 55, 69, …) and C_nH_{2n} series (m/z 28, 42, 56, 70, …) are of lower intensity. For alkenes the C_nH_{2n-1} (m/z 27, 41, 55, 69, …) series is most intense.[1] Thus comparing the intensities of the C_nH_{2n+1} and the C_nH_{2n-1} it is possible to distinguish roughly between alkanes and alkenes. In the GC-MS experiments mass spectra were recorded starting from m/z=45 and thus the pairs (55,56), (69,71), (83,85), (97,98) and (111,112) were compared. If in more than two of these pairs, the first mass showed a higher intensity, the compound was classified as an alkene. This classification was only used for peaks for which no reasonable library match was found in the NIST/EPA/NIH Mass Spectral Database (NIST 11) database. Otherwise, the library match was used.

Quantification of the product distribution in the condensed liquids was performed on a Varian 430-GC equipped with a FID detector and the same column as the GC-MS. The same sample dilution and column temperature profile was employed. The split ratio was set to 50 with an injection volume of 1 µl at a temperature of 265 °C. Using the same column for GC-MS and GC-FID allowed the use of identification from GC-MS for the quantification through GC-FID, assuming that the area fraction corresponds to the molar fraction. This assumption is justified as the signal of the FID mostly depends on the carbon content of the compound for hydrocarbons and thus directly to the molar weight. The chromatograms had to be aligned first. This alignment as well as peak detection and integration was

achieved using a customized python script. The same script was used to classify the compound as either aromatic if the name contained 'Benzene', 'Naphthalene', 'Chamazulene', 'Azulene', 'Xylene' or 'cumene'; as alkene if the name contained 'ene' and as alkane if the name contained 'ane'.

Infrared and Raman Spectroscopy

Fourier Transform-Infrared (FT-IR) spectra were collected in transmission as well as in attenuated total reflectance (ATR) mode on a PerkinElmer Frontier FT-IR spectrometer. For ATR, a Universal PerkinElmer ATR sampling accessory with a Diamond/ZnSe crystal and a MCT detector was used and spectra were recorded in a wavenumber range of 700 to 4000 cm^{-1} with a resolution of 1 cm^{-1} and adding 32 scans for each spectrum. The penetration depth is calculated according to $d_p = \lambda/(2 * \pi * n_c * \sqrt{\sin^2\theta - \left(\frac{n_s}{n_c}\right)^2}$ with refractive indices of the crystal $n_c = 2.4$ and PP $n_s = 1.49$.[42]

For performing transmission FT-IR spectroscopy, the samples were mixed with KBr or pressed pure into a self-supporting wafer. A $LiTaO_3$ detector was used and spectra were recorded in a wavenumber range of 675 to 4000 m^{-1} with a resolution of 1 cm^{-1} and adding 16 scans for each spectrum.

FT-IR spectroscopy with pyridine as probe molecule was performed in transmission mode on a ThermoScientific Nicolet iS5 instrument, equipped with a DTGS detector, using 32 scans per spectrum and a resolution of 8 cm^{-1}. Samples were prepared by pressing ~ 15 mg into a self-supported wafer, that was, subsequently, placed in a well-sealed cell with CaF_2 windows. Samples were dried at 400 °C (ramp of 10 °C/min) under high dynamic vacuum and kept at that temperature for 1 h. After cooling down to 50 °C, a spectrum was collected to identify the different hydroxyl groups present in the sample. Then, at RT, ~20 mbar of pyridine (Sigma-Aldrich, 99.8%) was allowed to adsorb on the sample for 30 min. Superfluous and loosely adsorbed pyridine was removed by evacuating the sample and keeping it under dynamic vacuum for 30 min after which the temperature was increased to 120 °C using a heating ramp of 5 °C/min. The final spectra were collected after keeping the spectra at 120 °C for 30 min.

Raman spectroscopy of the spent catalyst materials was performed on a Horiba Scientific XploraPlus Raman microscope equipped with a 638 nm laser operated at 1–10% laser power output, using a 1200 l/mm grating, and maximum 1×50 s exposure time.

Confocal Fluorescence Microscopy

Confocal fluorescence microscopy (CFM) was carried out on a Nikon Instruments A1 in DU4 mode using 403.7, 488, 561.1 and 641.5 nm lasers in combination with a 405/488/561/640 nm 1st dichroic mirror. Inverted sample stubs of the microtomed particles (**Figure S6**) were analyzed with both a Nikon LU Plan Fluor 10x/0.30 and Nikon LU Plan 50x/0.55 objective. This additional microtomy step was included due to the poor depth penetration of the laser light and subsequent fluorescence light as scattering and absorption significantly reduced the signal originating from the particle (**Figure S7**). For individual catalyst particle analysis, the 50x objective was used with a pixel dwell time of 21.6 ns/pixel, a raster size of 1024 pixels and a pinhole size of 66.4 µm. The laser power (LP) and detector sensitivity (HV) settings were kept the same for all images and chosen to utilize the full detector intensity range. The recorded fluorescence intensities were split across four bands of 425-475, 500-550, 570-620 and 663-738 nm and measured by a multi-anode photomultiplier. To analyze the microtomed catalyst particle particle images, the pixels were segmented into particle and background leading to a mask of the particle's cross section. The Euclidian distance between each pixel and the surface of the particle was calculated using the built-in MATLAB bwdist function. The obtained distance map was correlated

with the intensity of each of the detection channels normalized by their z-score with mean 0 and standard deviation 1 using the MATLAB normalize function.

Microtomy

The preparation of microtomy cross-sections is illustrated in **Figure S6**. A few catalyst particles were placed at the square-shape bottom of the microtomy vials, which were filled up with EpoFix resin. After drying at 120 °C for 12 hthe plastic of the vial was removed and the square-shaped top of the resin stub sliced and polished with a 35° or 45° DiATOME diamond knife in a Reichert-Jung Ultracut microtomy instrument.

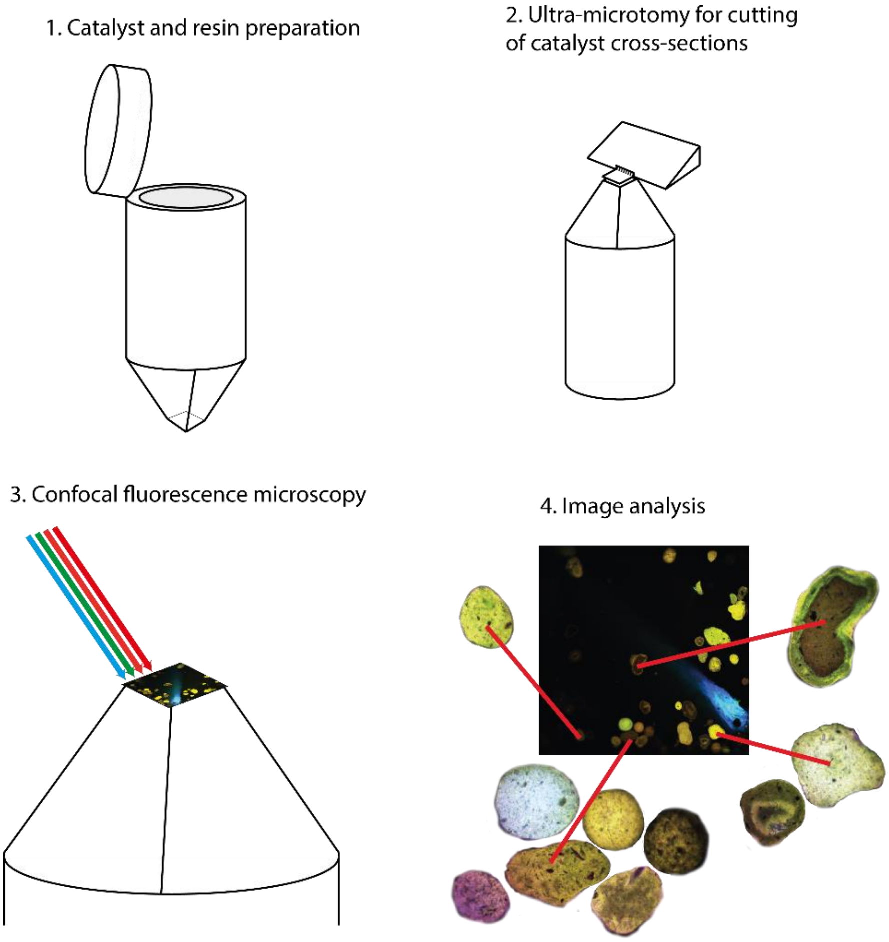

Figure S6. Schematic of the steps involved in creating the microtomy cross-sections for confocal fluorescence analysis.

Analysis of scattering effects in confocal fluorescence microscopy z-stack images

60 focal planes in z direction were scanned with a step length of 0.5 µm resulting a 3D image with four detection channels. The obtained images were segmented manually slice by slice classifying each voxel (analogous to a pixel in 3D) as either particle or background. This way, a 3D mask of each of the imaged particles was obtained. The distance d_i between each voxel i and the voxel with the lowest z-value with the same x,y coordinates in the 3D mask was computed using a MATLAB scrip. The intensity values of the four detection channels were correlated with the depth into material (d_i) (**Figure S7**). Due to adsorption and scattering effects, the intensity values decrease significantly with the depth into material.

Thermogravimetric analysis with online mass spectrometry

Catalysts recovered after reaction were analyzed using a PerkinElmer TGA 8000 coupled to a Hiden HPR-20 mass spectrometer to determine the total coke amount, the burn-off temperature and H/C ratio of the carbonaceous deposits. The about 5 mg of sample was heated to 800 °C (at a rate of 10 °C/min) under a 100 ml/min flow of artificial air (20 vol.% O_2 in Ar). The total mass loss was determined by taking the mass at 350 °C as the initial weight, assuming that any prior weight-loss is due to desorption of adsorbed water.

Impregnation of FCC-cat with Ni and Fe

Prior to impregnation, FCC-cat was dried at 150 °C under dynamic vacuum in a round bottom flask for 10 h. For incipient wetness impregnation (IWI) with Fe to achieve a final Fe weight loading of 2wt.% of Fe_2O_3, 2.94 g of FCC-cat was dried and impregnated with 152 mg of $Fe(NO_3)_2 \cdot 9(H_2O)$ dissolved in 380 µl of Mili-Q water by adding the solution dropwise under agitation with a glass rod. For IWI with Ni to aim at 3.75wt.% loading, 2.93 g of FCC-cat was dried. Subsequently 186 mg of $Ni(NO_3)_2 \cdot 9(H_2O)$ dissolved 526 µl of Mili-Q water was added under agitation with a glass rod. After impregnation samples were calcined byramping the temperature to 120 °C with 5 °C/min, holding the temperature for 20 minutes and subsequently ramping the temperature to 550 °C using a ramp of 10 °C/min and holding the temperature at 550 °C for 5 h.

Supplementary Results and Discussion

S1 Analysis of scattering effects in confocal fluorescence microscopy z-stack images

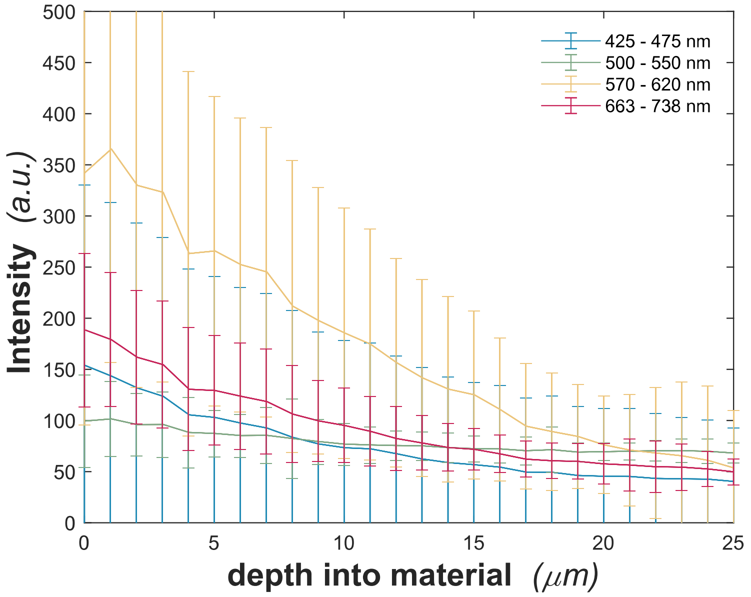

Figure S7. The intensity vs. depth into the particle analyzed from z-stack images of ECAT after full reaction under standard conditions.

S2 Pyridine FT-IR

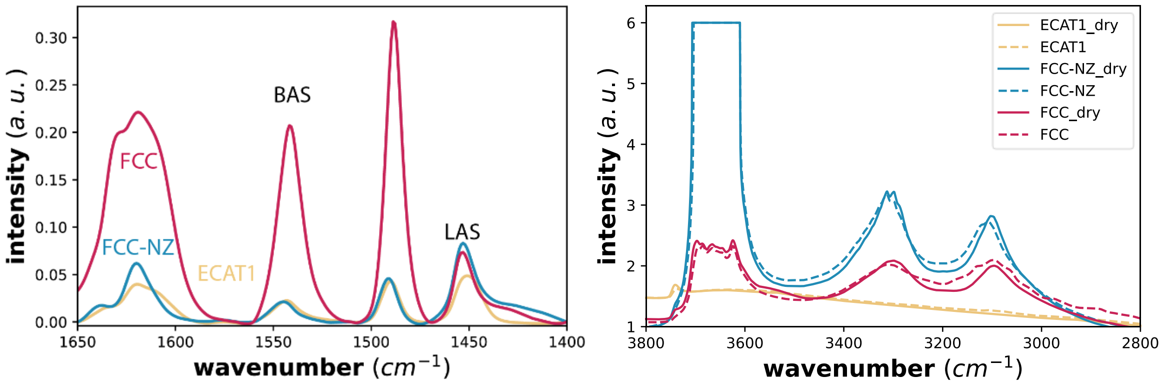

Figure S 8. Left: FT-IR spectra of pyridine stretching region after adsorption on Brønsted (BAS) and Lewis (LAS) acid sites. Right: FT-IR spectra of OH stretching region before (solid) and after (dashed) adsorption of pyridine.

S3 Ar physisorption results

Table S6. BET surface area, micropore volume and mesopore volume obtained from Ar physisorption on FCC-cat, ECAT and FCC-NZ.

	FCC	ECAT1	FCC-NZ	zeolite Y
BET surface area [m²/g]	261.1	183.2	83.6	872.9
micropore volume [µl/g]	84.0	49.4	3.9	225.2
mesopore volume [µl/g]	70.3	88.7	82.4	45.8

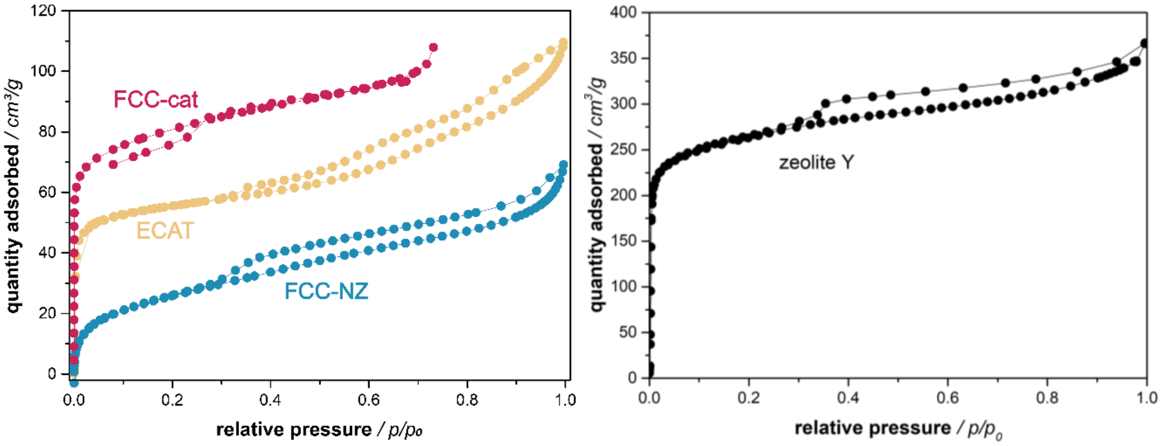

Figure S9. Ar adsorption isotherms of FCC-cat, ECAT and FCC-NZ (Panel A) and zeolite Y (Panel B).

S4 Product evolution

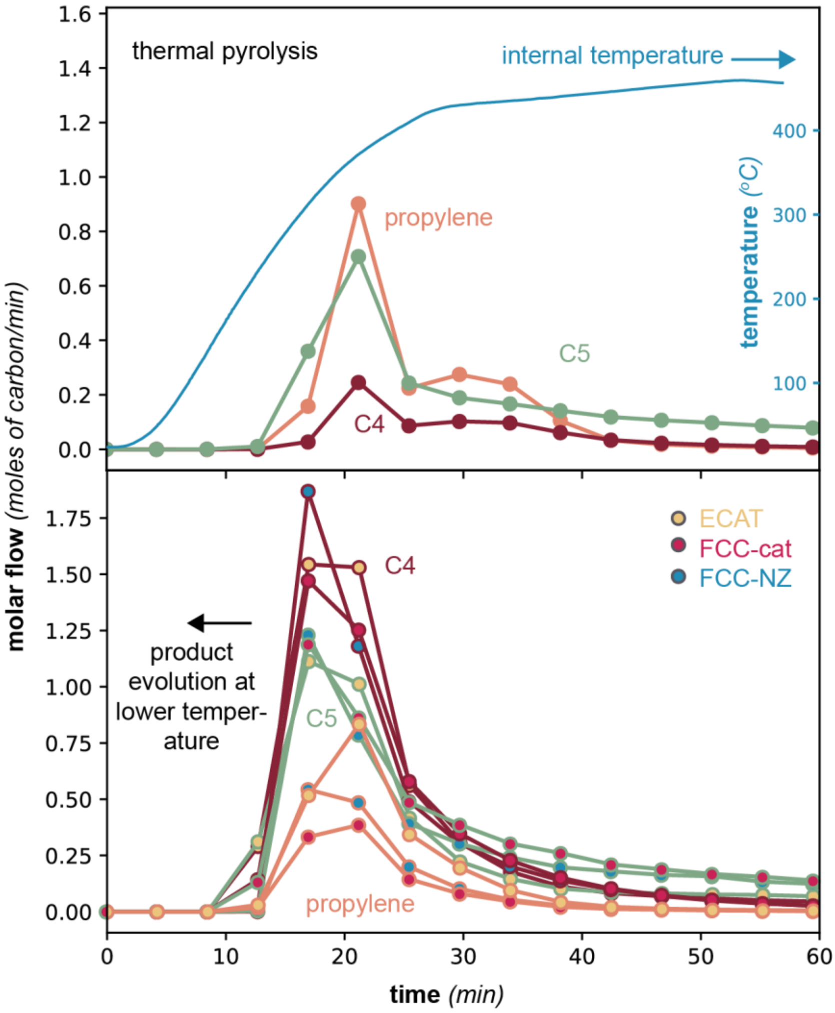

Figure S10. Molar flow of C3-5 products normalized by carbon number, measured over time of reaction and temperature in the reactor (heating rate 20 ℃/min) for reaction of 2.5 g PP without a catalyst (top) and with 1.25 g of either ECAT, FCC-cat or FCC-NZ (bottom).

S5 Analysis of condensable products

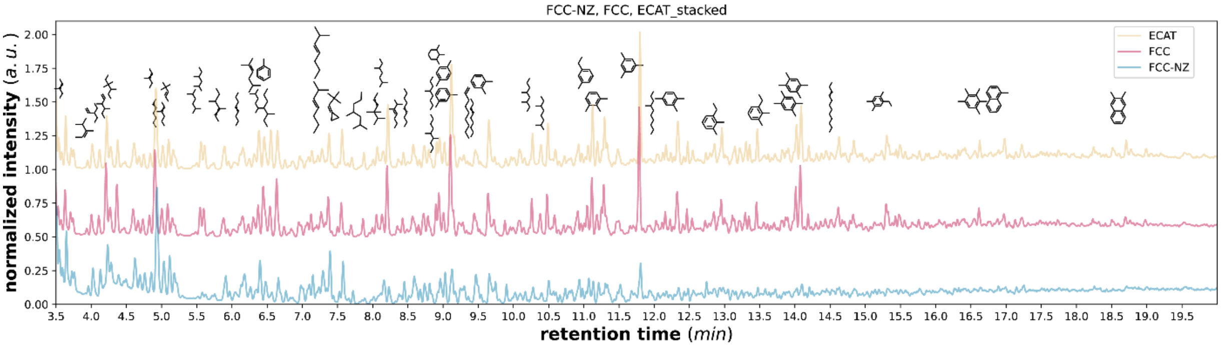

Figure S11. GC-MS chromatograms of condensable products measured after completion of the reaction in the presence of ECAT, FCC-cat and FCC-NZ.

S6 Hydrogen evolution profiles

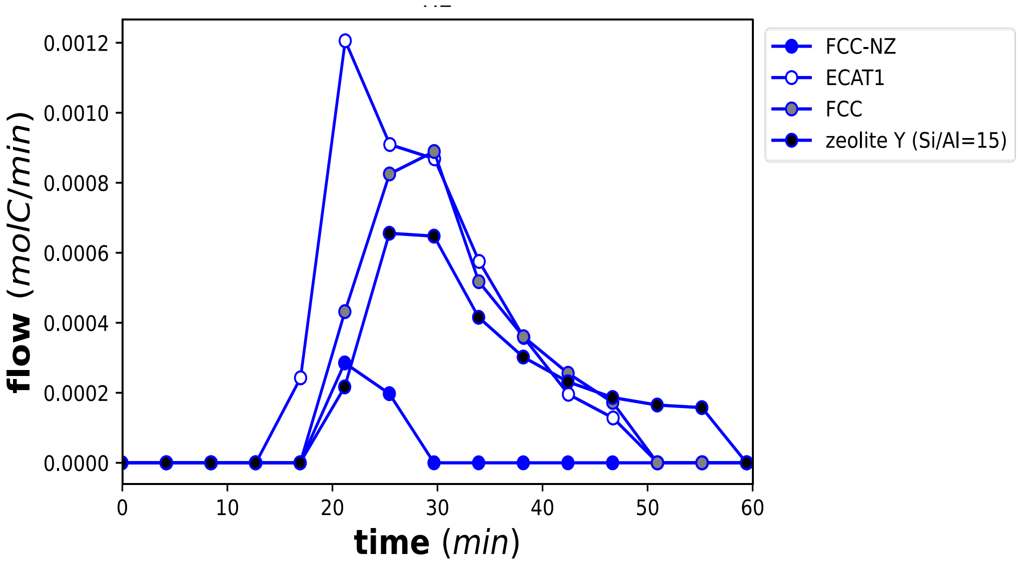

Figure S12. Hydrogen evolution with time on stream during reaction over FCC-NZ (filled symbols), ECAT (open symbols), FCC-cat (symbols with grey filling) and zeolite Y (symbols with black filling). Highest amounts of hydrogen are formed in the first 20 minutes of reaction over ECAT.

S7 Thermogravimetric analysis with online mass spectrometry

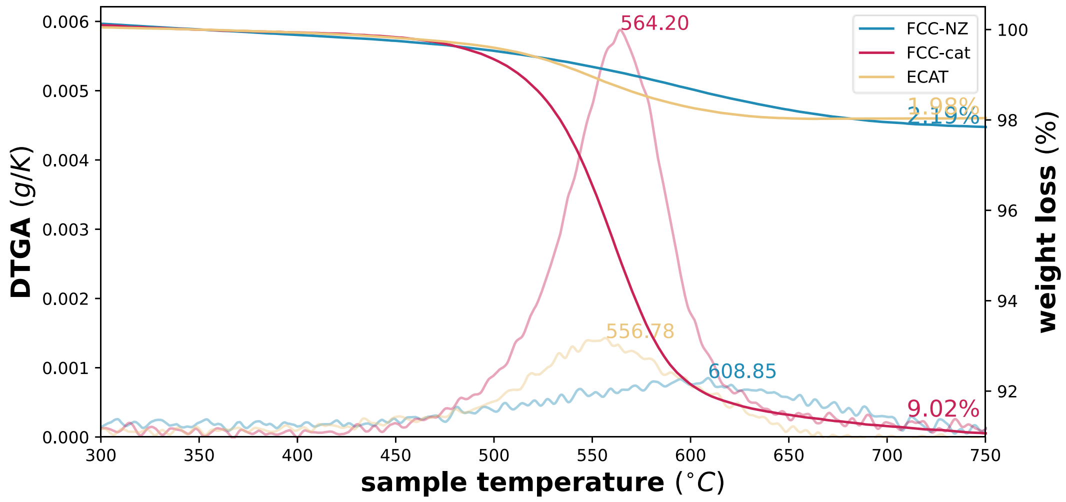

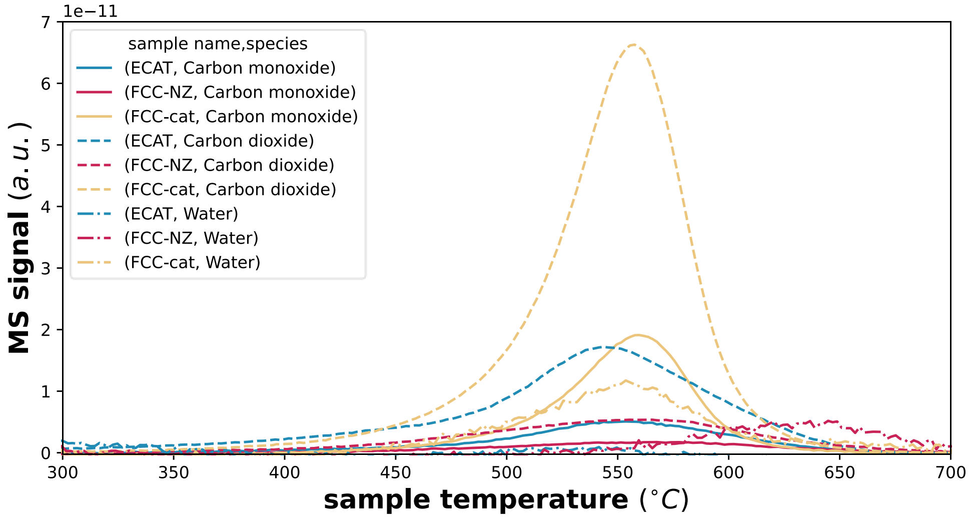

Figure S13. TGA and derivative TGA (DTGA) (Top) and MS traces of cabon monoxide, carbon dioxide and water (bottom) measured on the MS during TGA measurements of FCC-cat, ECAT and FCC-NZ after full reaction under standard conditions.

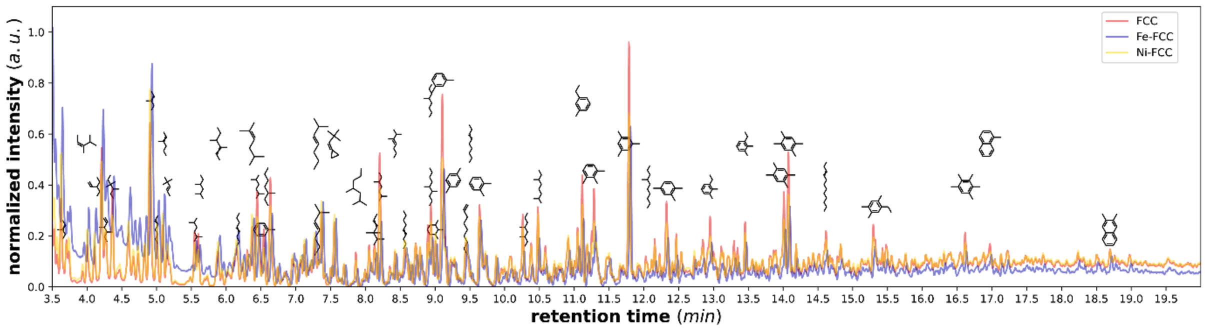

Figure S14. GC-MS chromatograms of condensable products measured after completion of the reaction in the presence of FCC-cat, Fe-FCC and Ni-FCC.

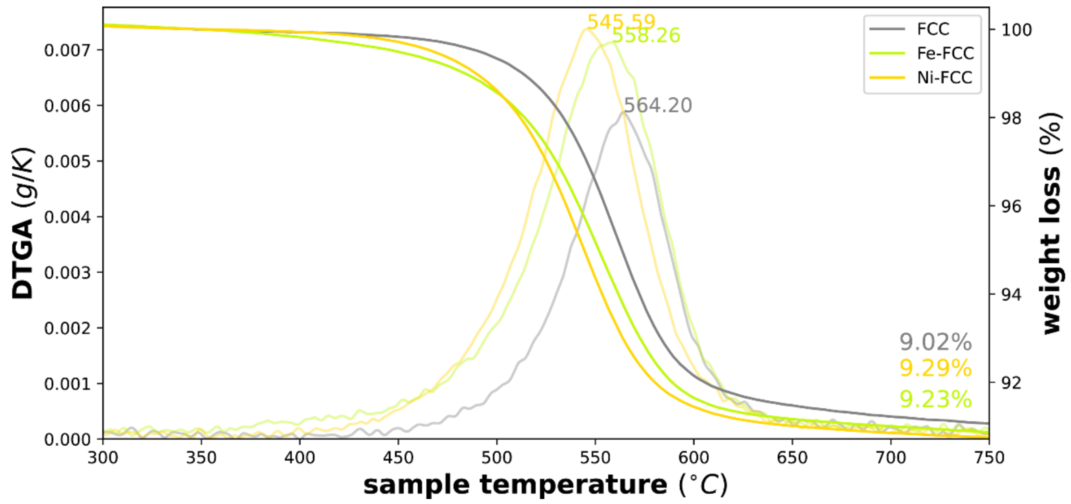

Figure S15. TGA and derivative TGA (DTGA) of FCC-cat and FCC-cat impregnated with Fe and Ni after full reaction under standard conditions.

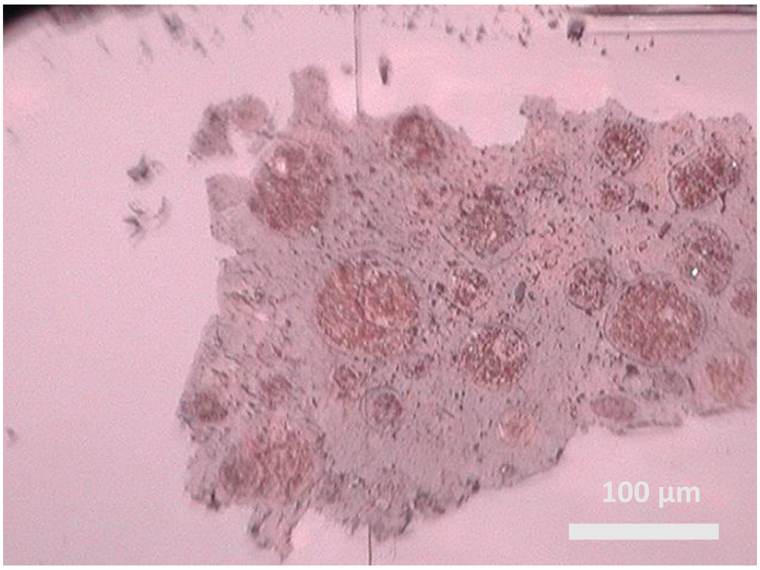

Figure S16. Microscopy image of ECAT embedded in partially cracked polypropylene as recovered after 13 min of reaction (250 ℃).

S8 Calculation of polymer chain length

The polymer chain length was calculated according to the following procedure.[2] The mean-square end-to-end distance was calculated using

$$\langle R^2 \rangle = C_\infty n l^2$$

Where C_∞ is the characteristic ratio defined by Flory as the ratio of the actual unperturbed mean-square end-to-end distance $\langle R^2 \rangle$ and that of a freely jointed chain nl^2. For isotactic polypropylene $C_\infty = 6.15$. n is the average number of repeat units in the polymer, calculated by dividing the average molecular weight M_w of polypropylene by the mass of a backbone bond of which there are two for each repeat unit and thus $M_b = 21.04 \frac{g}{mol}$ and $n = \frac{12{,}000 \frac{g}{mol}}{21.04 \frac{g}{mol}} = 570$ and l is the average bond length in the polymer and is 1.54 Å

The root mean square end-to-end distance becomes:

$$R_{rms} = \sqrt{\langle R^2 \rangle} = 91.21\ \text{Å} \approx 9\ nm$$

And the length of the fully extended chain:

$R_{max} = nl \cos\left(\frac{\theta}{2}\right) = 570 * 1.54\ \text{Å} * \cos\left(\frac{68°}{2}\right) = 728.17\ \text{Å} \approx 73\ nm$ with θ being the bond angle of the backbone bonds.

S9 Influence of polymer pellet size

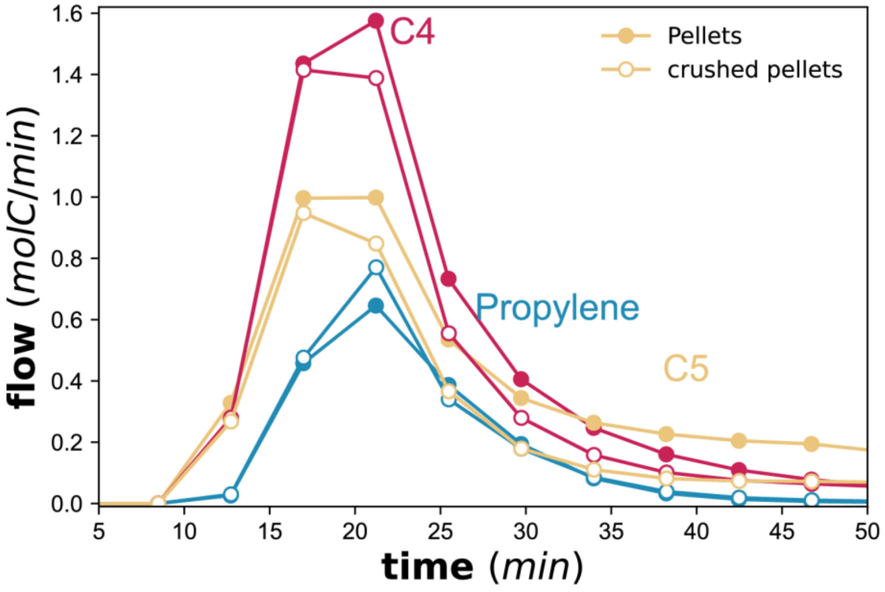

Figure S17. Molar flow of C3-5 products normalized by carbon number, measured over time of reaction and temperature in the reactor (heating rate 20 ℃/min) for reaction of 2.5 g of either as 2-8 mm PP pellets or the same pellets previously finely crushed over ECAT.

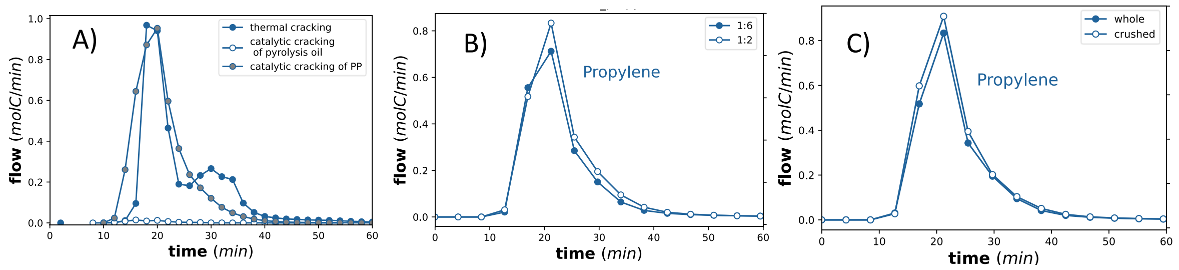

Figure S18. Propylene evolution profiles for A) thermal PP cracking and conversion of pyrolysis oil or PP over ECAT, B) Comparison of conversion of PP over 0.417 g (catalyst:polymer = 1:6) or 1.25 g of ECAT (catalyst:polymer = 1:2), C) Conversion over previously crushed ECAT catalyst and conversion over intact ECAT particles.

S10Two-step reaction

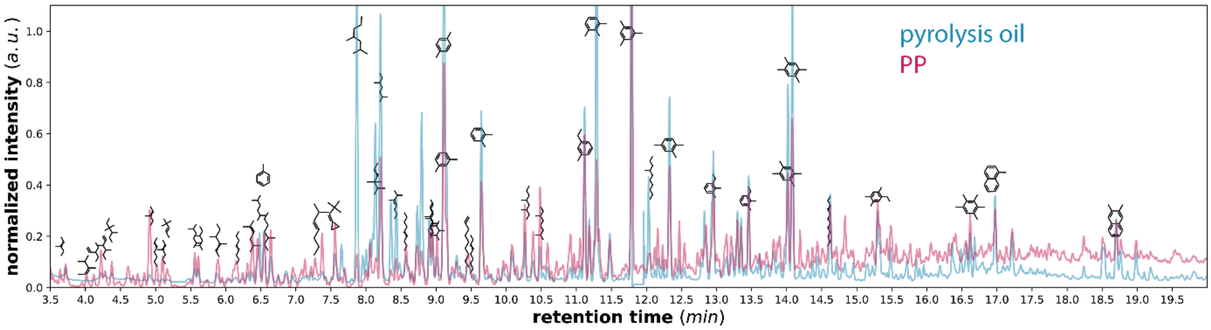

Figure S19. GCMS chromatograms of condensable products collected after catalytic runs of either 2.5 g of PP with 1.25 g of ECAT or 0.7 g of pyrolysis oil over 1.25 g of ECAT.

S11 Quenching the reaction after 13 min

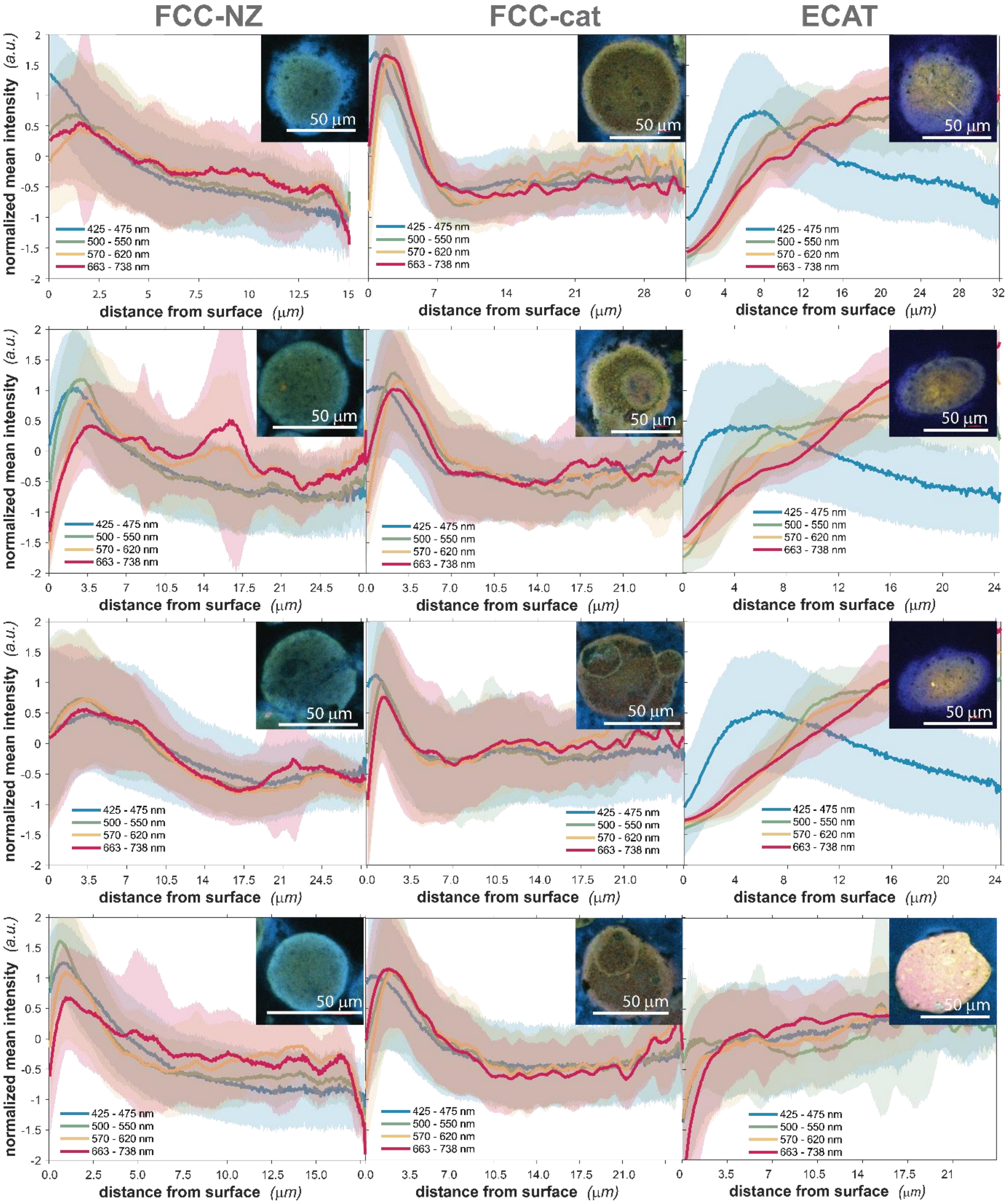

Figure S20. Radial intensity profiles of CFM images of microtomy cross-sectioned FCC-NZ (left column), FCC-cat (middle column) and ECAT (right column) particles after 13 min of reaction.

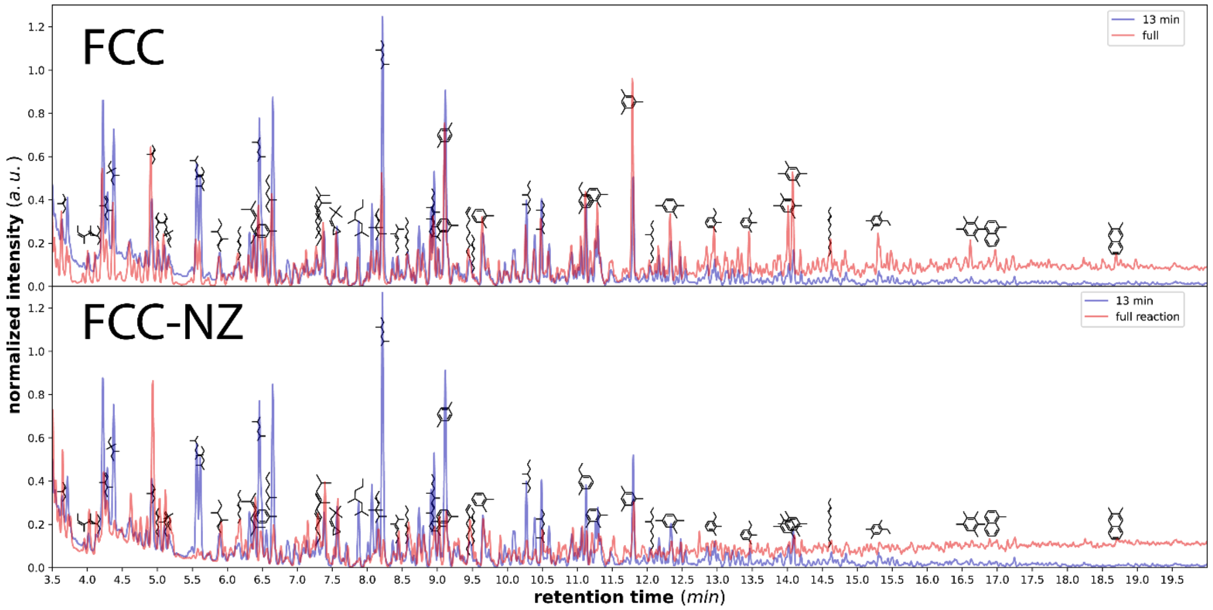

Figure S21. GC-MS chromatograms of the liquid products obtained after 13 min of reaction and after full reaction under standard conditions for FCC (top) and FCC-NZ (bottom).

S12 Effect of zeolite Y

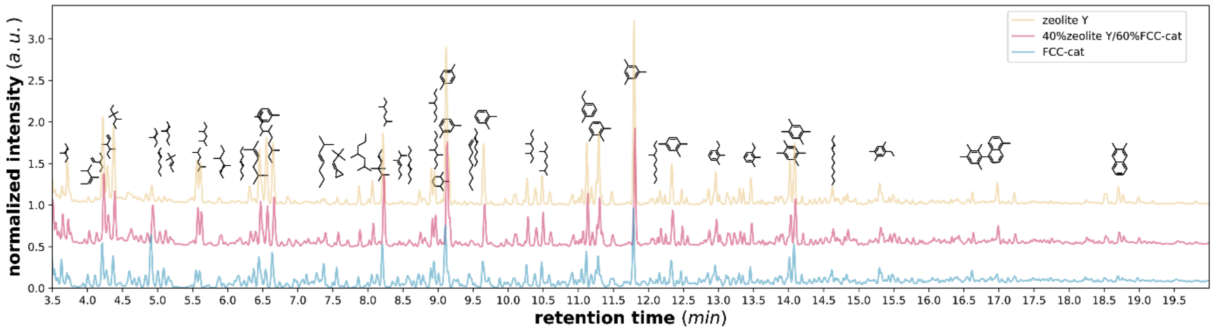

Figure S22. GC-MS chromatograms of the condensable products obtained after full reaction under standard conditions over FCC-cat (blue), a mixture of 40wt.% zeolite Y mixed with FCC-NZ (red) and zeolite Y (yellow).

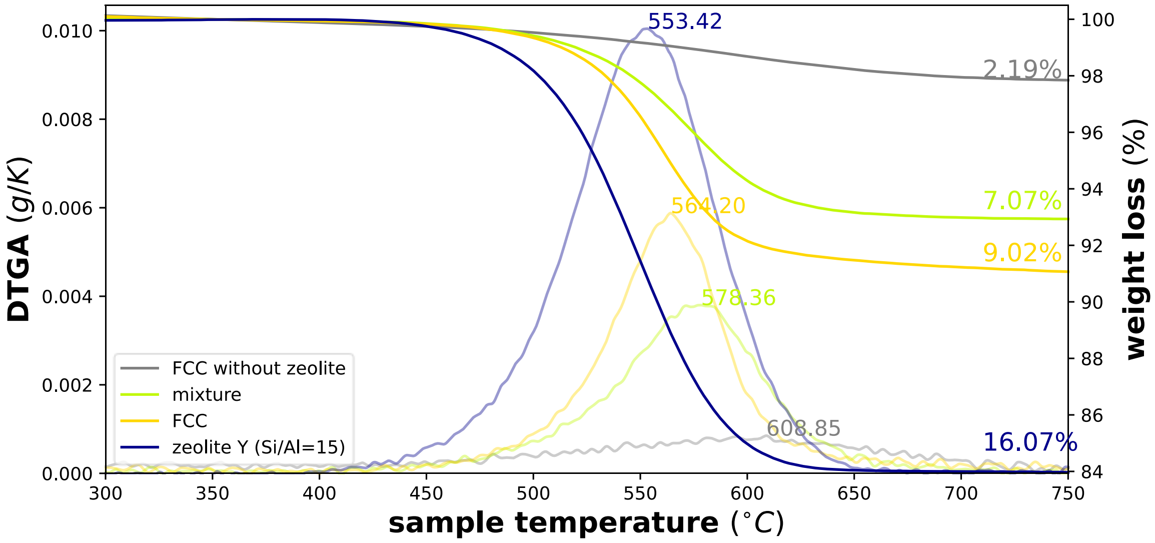

Figure S23. TGA and derivative TGA (DTGA) of FCC-NZ (grey), a mixture of 40wt.% zeolite Y mixed with FCC-NZ (green), FCC-cat (yellow) and zeolite Y (blue) after full reaction under standard conditions.

S13 References

[1] E. Pretsch, P. Bühlmann, C. Affolter, E. Pretsch, P. Bhuhlmann, C. Affolter, *Structure Determination of Organic Compounds*, Springer, Berlin, **2000**.

[2] L. J. Fetters, D. J. Lohse, R. H. Colby, in *Phys. Prop. Polym. Handb.*, Springer, Berlin, **2007**, pp. 447–454.
